# Use of cells and/or biologics combined with scaffolds in alveolar bone augmentation for implant site development

**DOI:** 10.1002/jper.70105

**Published:** 2026-09-14

**Authors:** Alberto Monje, Maria Costanza Soldini, Farahnaz Fahimipour, Manal Sarsri, Maria L. Geisinger

**Affiliations:** ^1^ Department of Periodontology and Oral Medicine University of Michigan Ann Arbor Michigan USA; ^2^ Department of Periodontology Universitat Internacional de Catalunya Barcelona Spain; ^3^ Department of Periodontology University of Bern Bern Switzerland; ^4^ Department of Periodontology University of Alabama Birmingham Alabama USA

**Keywords:** bone augmentation, bone graft, cell therapy, growth factor, maxillary sinus elevation, ridge augmentation

## Abstract

**Background:**

The impact of adjunctive biologics and the use of cell‐therapy has been sparsely explored. The objective of this systematic review is to evaluate the effectiveness/efficacy of these therapeutic modalities combined with scaffolds in alveolar bone augmentation for implant site development.

**Methods:**

An electronic and manual was conducted. Clinical trials evaluating the effectiveness/efficacy and safety of biologics and cell‐therapy combined with scaffolds used for bone alveolar augmentation procedures aiming at implant site development were eligible. The primary outcome was to assess the dimensional bone changes/gain, while the and secondary outcomes included histologic outcomes, patient‐reported outcomes, complications and adverse effects. Meta‐analysis was conducted only based on randomized clinical trials (RCTs). Moreover, risk of bias assessment of the selected investigations was also reported.

**Results:**

Overall, after screening, 68 articles were eligible for qualitative synthesis. Of these, 56 were selected of which 23 RCTs (n_patients _= 1078) were included in a quantitative analysis. In particular, six articles (n_patients _= 172) for lateral ridge augmentation, nine articles (n_patients _= 455) for alveolar ridge preservation, and eight articles (n_patients _= 451) for sinus floor elevation. All 23 RCTs explored the effectiveness/efficacy of adjunctive biologics. For ridge augmentation, the use of biologics resulted in superior outcomes in ridge width (RW) gain, especially the use of platelet rich plasma (*p* < 0.001), but not in ridge height (RH). For ridge preservation procedures, the adjunctive use of biologics rendered an added therapeutic benefit to maintain ridge dimensions, especially the use of bone morphogenetic protein‐2 for RW (*p* = 0.02) and RH (*p* = 0.09). Less non‐mineralized tissue was found based on histologic analysis for the use of biologics compared to control groups (*p* = 0.009). For sinus floor elevation, no benefit was shown in terms of RH gain (*p* = 0.87). However, upon histomorphometric analysis statistically less residual graft material was noted when biologics were used in combination with scaffolds (*p* < 0.001) compared to treatments without biologics. No complications or adverse events were found when using biologics or cell‐therapy in these applications. Use of the assessed adjunctive biologics generally did not have an influence on patient‐reported outcomes. Low risk of bias was demonstrated in the included studies.

**Conclusion:**

Findings from this review may suggest that biologics combined with scaffolds in alveolar ridge augmentation procedures may elicit superior outcomes in terms of dimensional bone gain and histological new bone formation compared to the use of grafts without applying biologics. Biologics and cell‐therapy are suggested to be safe and effective in alveolar bone augmentation interventions pursuing implant site development.

**Plain language summary:**

In recent years, researchers have been studying special biological products, called *biologics*, to help improve bone growth before placing dental implants. These products include things like growth factors or cells that can speed up healing and help new bone form. Our review demonstrates that when biologics are used together with bone graft materials, they appear to be safe and may lead to better bone growth and shape compared to traditional treatments. Patients also seem to prefer biologics especially when they come from their own blood because they may reduce pain and swelling after surgery. For procedures that lift the sinus to create more space for implants, using biologics did not show clear differences in bone height or appearance on X‐rays, although the bone healing process may change slightly when these products are used. Cell‐based therapies also seem safe and may work well, but there are not yet enough studies to prove they are better than traditional bone grafting methods.

## 1 INTRODUCTION

Insufficient or inadequate bone volume for ideal three‐dimensional (3D) implant positioning and long‐term peri‐implant health represents a common finding in clinical practice. Such deficiencies can be derived from different clinical conditions and biologic events taking place as consequence of tooth extraction, traumatism, resective surgical procedures, congenital and anatomic conditions, and secondary to periodontal and peri‐implant diseases. The presence of limited bone volume can compromise the ability to obtain optimal prosthetic design, peri‐implant health, function, and/or esthetics as well as the long‐term maintenance of the implant‐supported restorative fixtures. As such, implant site development (ISD) techniques are utilized to correct and overcome these limitations. ISD includes a variety of procedures that share aim at creating a recipient site adequate for implant placement in the optimal 3D position to achieve proper function, esthetics, and long‐term stability.^1^

Common hard tissue ISD procedures include: alveolar ridge preservation, horizontal and vertical ridge augmentation, and maxillary sinus floor elevation.^2^ These can be performed with a variety of techniques and materials, and each demonstrates clinical indications and limitations. Absorbable and non‐absorbable membranes, particulate grafting materials from different origins, and autologous and/or allogeneic bone blocks are among the most frequently employed techniques/materials for hard tissue ISD reconstruction.^2^ While these techniques demonstrate relatively high success rates in multitude of investigations, such grafting protocols present with drawbacks and limitations, including, but not limited to, challenges during the healing phase, reduced mineralized tissue formation compared to native bone, infection risk, poor graft volumetric stability, extended healing time, limitations of reconstructive bone volume endpoints, and so forth. In order to overcome these limitations and enhance bone healing outcomes, the adjunctive use of biologics has been proposed.^3^

Biologics are therapeutic agents with biological activity that are administered to achieve an enhanced regenerative or reparative effect. These materials can promote a variety of essential cellular and molecular events critical to wound healing, including deoxyribonucleic acid (DNA) synthesis, chemotaxis, cell differentiation, mitogenesis, neovascularization, cell‐to‐cell communication, and matrix biosynthesis. Consequently, these biologics have been used to enhance hard tissue regeneration outcomes by means of improving the inflammatory resolution, reducing the post‐operative pain and sequelae, leading to a more rapid wound closure, and improving the new bone formation.^3^ Moreover, cell‐therapy, including the use of bone‐derived mesenchymal stem cells, has been used in orthopedic and craniofacial regeneration and has demonstrated safety and effectiveness for the reconstruction of a variety of lost tissues.^4^

While the use of biologics in implant therapy for ISD interventions has been extensively studied,^1^, ^3^, ^4^, ^5^, ^6^, ^7^, ^8^ clinical evidence in this context is growing in quantity and quality over the last few years. Moreover, the impact of cell‐therapy in conjunctive with traditional ISD procedures has been sparsely explored. The aim of the present review was to identify and assess existing scientific evidence from experimental, non‐experimental, observational comparative, case‐control and/or case series clinical studies that tested the safety, effectiveness, and/or efficacy of biologics and/or cell‐therapy combined with scaffolds in alveolar bone augmentation procedures preceding dental‐implant placement. Further, we aimed to conduct a systematic review and, if possible, perform meta‐analyses.

## 2 MATERIALS AND METHODS

The review protocol was approved on November 4th, 2024 (identification number CRD CRD42024605421) in the PROSPERO International Prospective Register of Systematic Reviews hosted by the National Institute for Health Research, University of York, Centre for Reviews and Dissemination. For describing and summarizing the results of the review, we employed the 27‐item PRISMA (Preferred Reporting Items for Systematic Reviews and Meta‐Analyses) statement.^9^ This review focused on the following focused and PICO questions:

### 2.1 Focused question 1

Does the utilization of biologics in combination with scaffolds (test) provide with superior, equal or inferior outcomes when compared to control/comparative interventions for bone augmentation with regard to clinical, radiographic, imaging, histologic, peri‐operative outcomes, and patient‐reported outcomes (PROs) of ISD procedures?

### 2.2 Focused question 2

Does the utilization of cell‐therapy in combination with scaffolds (test) provide superior, equal, or inferior outcomes when compared to control/comparative interventions for bone augmentation with regard to clinical, radiographic, imaging, histologic, peri‐operative outcomes, and PROs of ISD procedures?

### 2.3 PICOS question 1

**P**: Patients presenting bone defects in the jaws requiring bone augmentation procedures prior or simultaneous to implant placement

**I**: Performance of ISD using the following biologics: enamel matrix derivatives, bone morphogenetic proteins, autologous blood‐derived products, and platelet‐derived growth factor, combined with a scaffold without barrier membrane or guided bone regeneration (GBR) using resorbable or non‐resorbable barrier membrane procedure performed simultaneously or staged to implant placement. Other biologics not currently being broadly commercialized (i.e., growth and differentiation factor‐5) were excluded.

**C**: Performance of ISD using scaffold without barrier membrane or GBR using resorbable or non‐resorbable barrier membrane procedures without biologics

**O**:

- Primary outcomes: Vertical and/or horizontal bone gain (radiographically or clinically) of residual defects for simultaneous implant placement scenarios and vertical and/or horizontal bone gain (radiographically or clinically upon surgical reentry) for staged implant placement.
- Secondary outcomes: Dental implant placement feasibility (suitable site for implant placement in the planned position) and implant stability (insertion torque or implant site development), implant survival, biological complications, adverse effects, PROs, clinician‐reported outcomes (CROs) healing time, histomorphometric analysis (i.e., new bone formation in staged approaches), subjective bone quality and density as assessed by the clinician.

**S**: Randomized or non‐randomized clinical trials with parallel‐arm or split‐mouth design, prospective single‐arm case series

### 2.4 PICOS question 2

**P**: Patients presenting bone defects in the jaws requiring bone augmentation procedures prior to implant placement

**I**: Performance of ISD using cell‐therapy combined with without barrier membrane a scaffold or GBR (using resorbable or non‐resorbable barrier membrane) procedure staged to implant placement

**C**: Performance of ISD using scaffold without barrier membrane or GBR (using resorbable or non‐resorbable barrier membrane) procedures without cell‐therapy

**O**:

- Primary outcomes: Vertical and/or horizontal bone gain (radiographically or clinically) of residual defects for simultaneous implant placement scenarios and vertical and/or horizontal bone gain (radiographically or clinically upon surgical reentry) for staged implant placement.
- Secondary outcomes: Dental implant placement feasibility (suitable site for implant placement in the planned position) and implant stability (insertion torque or implant site development), implant survival, biological complications, adverse effects, PROs, clinician‐reported outcomes (CROs) healing time, histomorphometric analysis (i.e., new bone formation in staged approaches), subjective bone quality and density as assessed by the clinician.

**S**: Randomized or non‐randomized clinical trials with parallel‐arm or split‐mouth design, prospective single‐arm case series

### 2.5 Eligibility criteria

Articles screened for inclusion in this review met the following inclusion and exclusion criteria:

‐ Inclusion criteria:
  - Randomized or non‐randomized clinical trials with parallel‐arm or split‐mouth design, prospective single‐arm case series
  - ≥10 subjects per arm
  - Systemically healthy subjects
  - Including re‐assessment by any means to test bone dimensional changes, histological outcomes, PROs and adverse events
  - No limitation in terms of follow‐up following the ISD intervention was applied.
‐ Exclusion criteria:
  - Retrospective or cross‐sectional cohort, case series or case report studies
  - Inclusion of heavy smokers ≥10 cig/day
  - Presence of, or lack of assessment for, systemic disease that could impact bone healing

### 2.6 Information sources and literature search strategy

Electronic and manual literature searches were conducted independently by two reviewers (AM, MS) in several databases, including MEDLINE (OVID), EMBASE (OVID), Cochrane Central Register of Controlled Trials (Cochrane Library), and Cochrane Oral Health Group Trials Register (Cochrane Library), databases for reports published up to November 1, 2024, without language restrictions. Additionally, the ‘grey literature’ at the New York Academy of Medicine Grey Literature Report (http://greylit.org) and the register of clinical studies hosted by the US National Institutes of Health (www.clinicaltrials.gov) were searched to further identify potential literature candidates for inclusion. Bibliographies of identified candidate articles were also searched for further identification of potential articles for inclusion. The reviewers independently extracted predetermined data from the included reports (M.S., C.S.). In cases of missing or equivocal data deemed pivotal to the analyses, attempts were made to contact the corresponding authors of the primary studies. Where contact was not achieved or in other cases of missing data information was recorded as not reported (N.R.) in the data set.

### 2.7 Electronic literature searching

A comprehensive electronic search was performed in PubMed, the Cochrane Library, and EMBASE to identify human studies published up to and including November 1, 2024. The search was restricted to studies conducted in humans and published in the English language. The detailed search strategies for each database are provided in Supplementary Table S1.

### 2.8 Methodological quality and risk‐of‐bias assessment

The methodological quality of the included studies was assessed using tools appropriate to their study design. For randomized controlled trials, we applied the Risk of Bias 2 tool (RoB 2, 2019 version), which is specifically designed to evaluate potential bias across domains such as the randomization process, deviations from intended interventions, missing outcome data, outcome measurement, and selection of the reported results.^10^ For non‐randomized studies, we used the ROBINS‐I tool, version 2, which provides a structured framework for assessing bias in studies that do not employ random allocation, including potential confounding, selection bias, classification of interventions, deviations from intended interventions, missing data, measurement of outcomes, and selection of the reported results.^11^

### 2.9 Data synthesis

Data were gathered into evidence tables and presented according to the objective/indication of the surgical procedure of interest. The feasibility of running pooled estimates was considered based on the data of included studies by an independent biostatistician. Moreover, based on the available data and results of this systematic review, critical assessment of the literature and evidence quality rating or strength of recommendation of biologics, for implant‐site development procedures, were applied. These recommendations were based on the criteria established by the adapted version of the American Dental Association (ADA) Clinical Practice Guidelines^12^ in which biologics and cell‐based surgical treatment modalities displayed in this review were presented to provide data to assist the interpretation of outcomes. Moreover, relevant adverse events and complications were retrieved to assess whether the expected benefits outweigh the potential for harm. PROs were also reported. In addition, conflict of interest was extracted from the studies and reported in Supplementary Table S2.

Findings were deemed suitable to conduct meta‐analyses to evaluate outcomes in PICO question 1. The primary focus of the meta‐analyses was comparison of retrievable outcomes reported in mean value and standard deviation between the test and control groups for the procedures included, that is, ridge augmentation, alveolar ridge preservation, and maxillary sinus floor elevation. Mean differences of outcomes between test and control groups were estimated for each study and the weighted mean difference (WMD) was reported as the global effect measure in a random‐effects model with corresponding *Z*‐statistics, *p*‐values, and 95% confidence intervals. Regarding heterogeneity analysis, Cochran's *Q* test was applied. *I* ^2^ index was also calculated, representing the amount of between‐studies variability compared to total variability. Expressed as a percentage, *I* ^2^ ranges from 0% to 100%. Cutoff points 25%, 50%, and 75% are associated to low, medium and high levels of heterogeneity. Funnel graphs were also performed to assess the potential publication bias. Egger's tests were applied to contrast this hypothesis. In addition, whenever possible, subset analyses were conducted for articles testing biologics combined with scaffolds to test the effectiveness of the individual biologics on treatment outcomes. Meta‐analysis was deemed infeasible for cell‐based therapies due to the limited sample size and data heterogeneity. Additionally, network meta‐analysis (NMA) was not performed as it was determined that the variable composition of the control group for each article was not amenable to allow for a validated NMA. The level of significance used in the analysis was 5% (*α* = 0.05).* The software used were packages Metafor from R 4.3.1 (R Core Team (2023). R: A language and environment for statistical computing. R Foundation for Statistical Computing, Vienna, Austria. URL: http://www.R‐project.org/).

## 3 RESULTS

**FIGURE 1:**
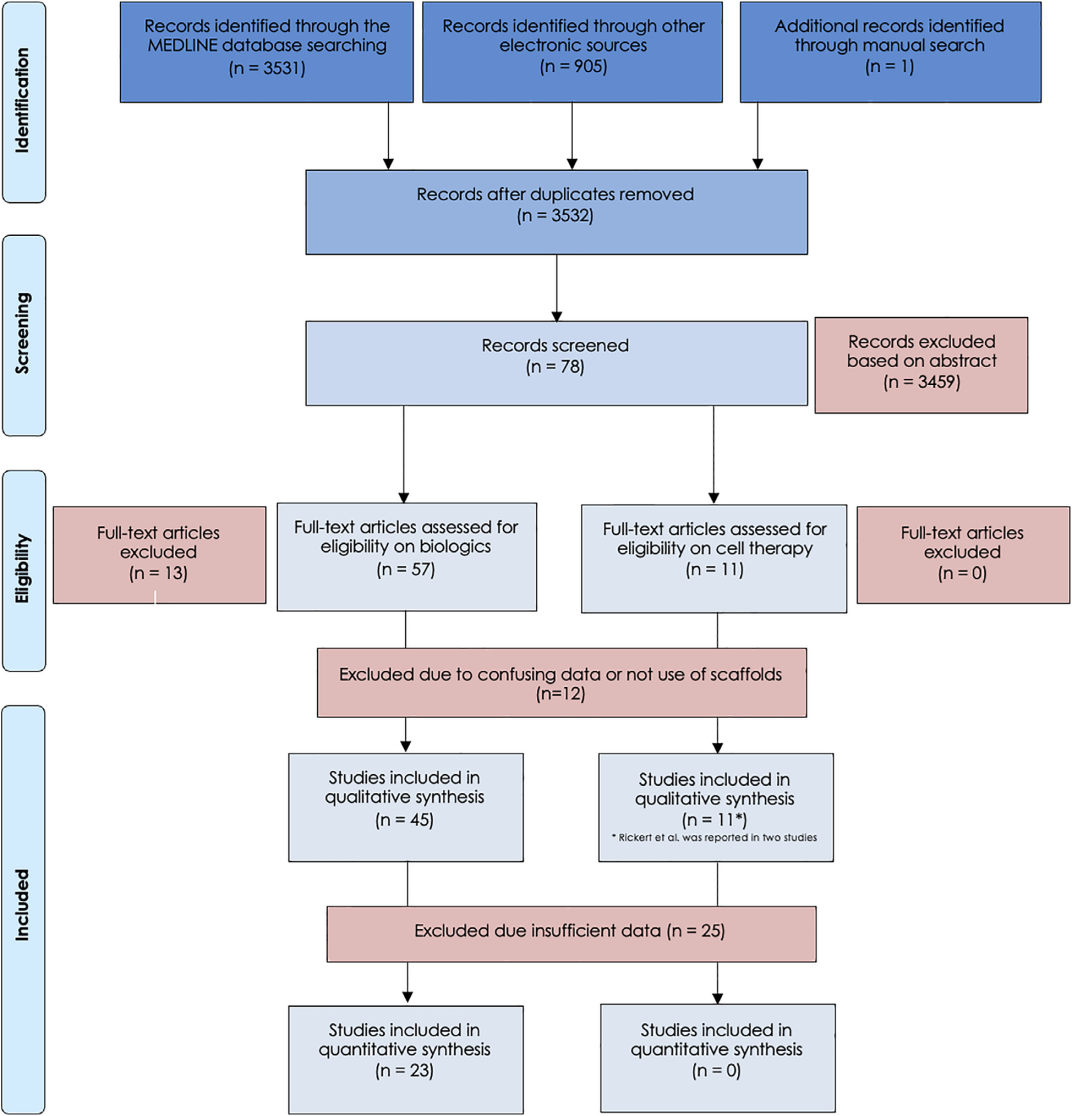
Flow chart of the screening process.

The PRISMA flowchart for literature selection is depicted in Figure 1. In summary, 3532 records were identified after duplicates removed. Moreover, nine additional records were identified within the bibliographies of included/excluded papers, for a total of 3541 records evaluated. Of these, 80 were assessed for full‐text and 66 were included in qualitative analysis.^13^, ^14^, ^15^, ^16^, ^17^, ^18^, ^19^, ^20^, ^21^, ^22^, ^23^, ^24^, ^25^, ^26^, ^27^, ^28^, ^29^, ^30^, ^31^, ^32^, ^33^, ^34^, ^35^, ^36^, ^37^, ^38^, ^39^, ^40^, ^41^, ^42^, ^43^, ^44^, ^45^, ^46^, ^47^, ^48^, ^49^, ^50^, ^51^, ^52^, ^53^, ^54^, ^55^, ^56^, ^57^, ^58^, ^59^, ^60^, ^61^, ^62^, ^63^, ^64^, ^65^, ^66^, ^67^, ^68^, ^69^, ^70^, ^71^, ^72^, ^73^, ^74^, ^75^, ^76^, ^77^, ^78^ Overall, 31 articles (n_patients _= 1369) met the initial criteria for the quantitative analysis. However, eight were excluded after further review due to the lack of scaffold employment in combination with biologics. The remaining articles were grouped according to the surgical aim of the biologic or cell therapy, namely, ridge augmentation, ridge preservation, maxillary sinus floor elevation, and implant placement. Hence, 54 studies were included in qualitative synthesis, while 23 RCTs (n_patients _= 1078) met the criteria and provided sufficient data to be meta‐analyzed. In particular, six articles (n_patients _= 172) for ridge augmentation, nine articles (n_patients _= 455) for alveolar ridge preservation, eight articles (n_patients _= 451) for maxillary sinus floor elevation were included in this systematic review. An updated manual screening performed September 21, 2025, unveiled two studies more that provided data for qualitative analysis of cell therapy, which amounted to 56 the studies included in qualitative analysis.^79^, ^80^

### 3.1 Ridge augmentation

#### 3.1.1 Dimensional changes

**FIGURE 2:**
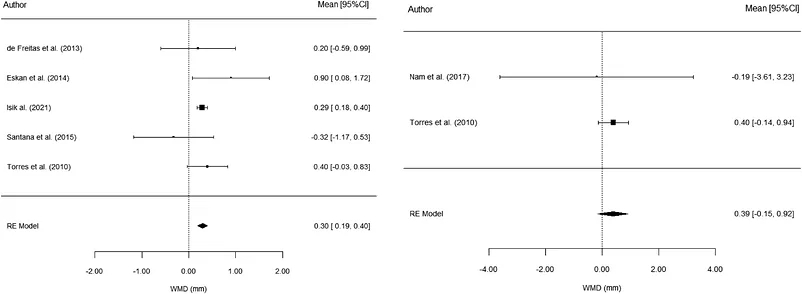
Meta‐analysis showing weighted mean differences (WMD) for the outcomes ridge width (left panel) and ridge height (right panel) for ridge augmentation.

For biologics, in terms of ridge width (RW) changes, meta‐analysis based on five studies^27^, ^30^, ^39^, ^63^, ^72^ demonstrated a WMD = 0.30 mm. Statistical significance was reached (*p* < 0.001), demonstrating benefit of biologics used in combination to scaffolds compared to control groups. The heterogeneity was estimated at a null level (*I* ^2 ^= 0%). Moreover, the Funnel plot suggested high level of symmetry (*p* = 0.916, Egger's test). Given the representativeness of platelet‐rich plasma (PRP), meta‐analysis based on two studies^30^, ^72^ demonstrated a WCD = 0.52, suggesting the outperformance (*p* = 0.015) of this biologic to control groups in RW gain. The heterogeneity estimated was relatively low (*I* ^2 ^= 11%). Moreover, two studies^57^, ^72^ reported on ridge height (RH). Meta‐analysis suggested higher RH gain for test groups (WMD = 0.39); nevertheless, statistical significance was not reached (*p* = 0.155) (Figure 2). The heterogeneity was estimated at a null level (*I* ^2 ^= 0%).

For cell‐therapy, four studies^36^, ^45^, ^46^, ^79^ explored ridge augmentation. Meta‐analysis was determined not to be feasible. Stem‐cells seeded on a synthetic bioabsorbable bone graft when compared to autogenous blocks outperformed in terms of volume gain (*p* = 0.03).^79^ Otherwise, no noticeable superiority of cell‐therapy combined with scaffolds in contrast to scaffolds alone was demonstrated in other included studies.^45^, ^46^

#### 3.1.2 Histologic outcomes

For biologics, meta‐analysis was not feasible due to limited data reporting on histologic performance of adjunctive biologic use.

For cell‐therapy, none of the included studies reported on histologic outcomes.

#### 3.1.3 Adverse events and complications

For biologics, four studies^27^, ^37^, ^71^, ^72^ reported complications associated to this intervention. There was no evidence reporting major complications for either test or control groups. Higher facial swelling was noticed when recombinant human bone morphogenetic protein‐2 (rhBMP‐2) was used compared to the use of scaffolds without biologics. No major adverse events were reported.

For cell therapy, only two studies^36^, ^46^ reported on peri‐operative adverse events. No major post‐operative complications were reported. More dehiscence's were reported for the application of guided bone regeneration using stem cells when compared to the control group.^46^ However, statistical significance was not reached.

#### 3.1.4 Patient‐reported outcome measures

For biologics, two studies^37^, ^70^ reported on PROs. Reduced post‐operative discomfort was reported when platelet‐rich fibrin (PRF) was used compared to other treatment modalities. Further, intra‐ and extra‐oral sensitivity at suture removal and 4‐month follow‐up was greater for the use of rhBMP‐2 and the willingness to repeat the surgery was lower for the control group. No major adverse events were reported.

For cell‐therapy, only two studies^36^, ^46^ reported on PROs. In general, these studies reported a high level of patient satisfaction and willingness to recommend the intervention to others due favorable outcomes.

#### 3.1.5 Quality assessment

Quality assessment for each of the included studies was based on the RoB 2 tool for assessing the risk of bias for RCTs. Most of the included studies revealed mid to low risk of bias (Supplementary Figure S1). The majority of the studies exhibit some concerns regarding the reporting of results. Common concerns included absence of registration in the ClinicalTrials.gov database and omission of information on complications, among other limitations.

### 3.2 Ridge preservation

#### 3.2.1 Dimensional changes

**FIGURE 3:**
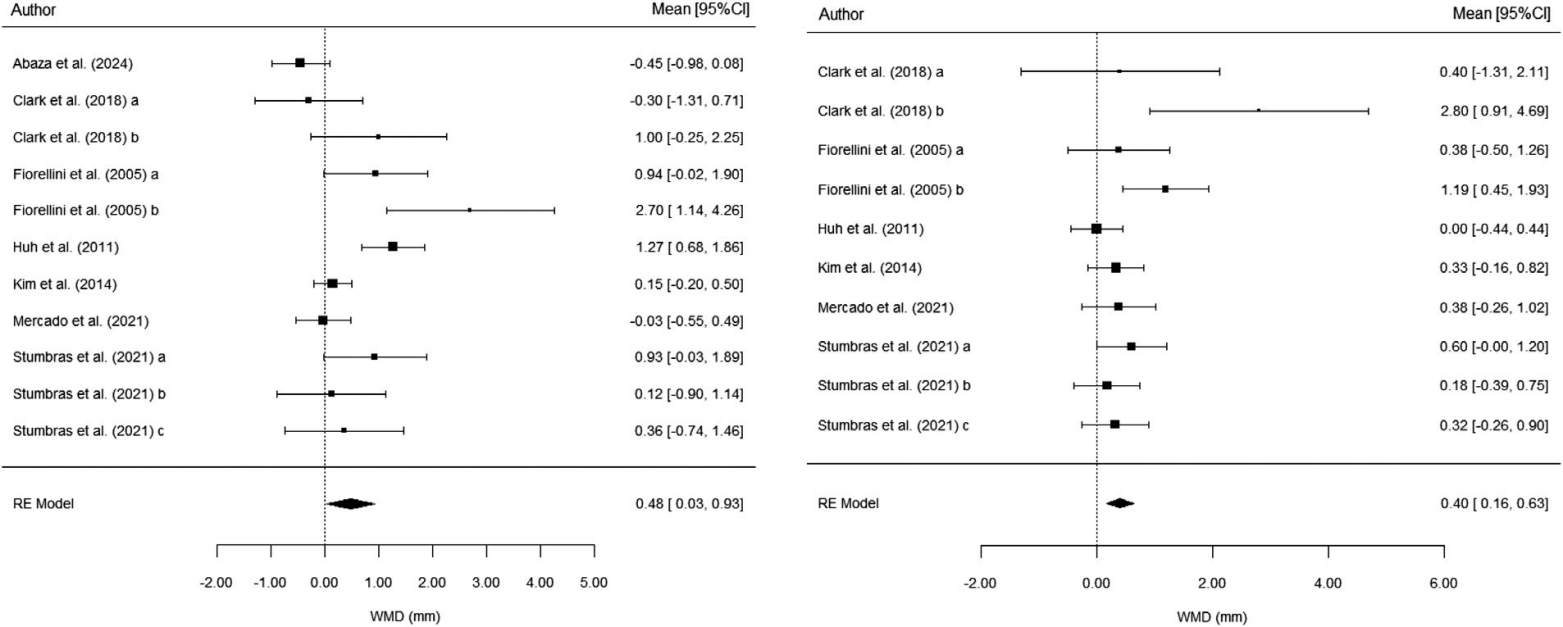
Meta‐analysis showing weighted mean differences (WMD) for the outcomes ridge width (left panel) and ridge height (right panel) for ridge preservation.

For biologics, in terms of RW, meta‐analysis based on seven studies^13^, ^24^, ^32^, ^38^, ^48^, ^53^, ^65^ demonstrated a WMD = 0.48 reaching statistical significance (*p* = 0.035). This demonstrated a benefit for the adjunctive use of biologics compared to control groups. Heterogeneity was estimated at a moderate‐high level (*I* ^2 ^= 74%). The Funnel plot showed some level of asymmetry (*p* = 0.054, Egger's test). In addition, meta‐analysis^32^, ^38^, ^48^ was performed to test the effect of rhBMP‐2 on RW. Meta‐analysis demonstrated WMD = 1.08 mm, suggesting a statistically significant enhanced outcome for the test groups (*p* = 0.02). Heterogeneity was estimated at a high level (*I* ^2 ^= 84.6%). Further, six studies^24^, ^32^, ^38^, ^48^, ^53^, ^65^ reported on RH. Interestingly, meta‐analysis demonstrated a WMD = 0.40, suggesting strong statistical significance favoring test groups versus control procedures (*p* < 0.001) (Figure 3). Heterogeneity was estimated as irrelevant (*I* ^2 ^= 5%). However, a possible publication bias was detected (*p* = 0.010, Egger´s test). Marginal statistical significance was reached demonstrated a benefit of rhBMP‐2 on RH based on three studies^32^, ^38^, ^48^ (WMD = 0.42; *p* = 0.09).

For cell‐therapy, only one study^42^ reported on dimensional bone changes when compared to guided bone regeneration. At 6‐weeks, but not 12‐weeks, cell therapy demonstrated statistically significant improvements in linear ridge height compared to conventional guided bone regeneration. Moreover, when early re‐entry was performed for implant placement sites that received cell‐therapy demonstrated a tendency towards a reduction in simultaneous bone grafting required for implant stabilization.

#### 3.2.2 Histologic outcomes

**FIGURE 4:**
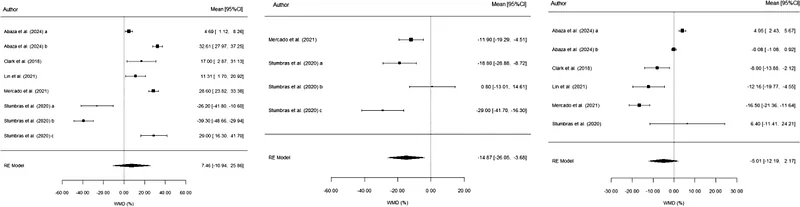
Meta‐analysis showing weighted mean differences (WMD) for the histologic outcomes % mineralized tissue (left panel), % non‐mineralized tissue (middle panel), and % residual graft (right panel) for ridge preservation.

For biologics, five studies^13^, ^24^, ^51^, ^53^, ^66^ reported on percentage of mineralized tissue. Meta‐analysis demonstrated a WMD = 7.46%. Statistical significance was not reached (*p* = 0.427). Heterogeneity was estimated as very high (*I* ^2 ^= 98%). No publication bias was detected (*p* = 0.379, Egger's test). Higher % of non‐mineralized tissue was noticed for control groups (WMD = −14.9%), reaching statistical significance (*p* = 0.009). This analysis was based on two studies.^53^, ^66^ Heterogeneity was estimated as moderate (*I* ^2 ^= 77%). No publication bias was claimed (*p* = 0.893, Egger's test). The analysis on the % of residual graft was based on five studies.^13^, ^24^, ^51^, ^53^, ^66^ No statistical significance was reached between test and control groups (WMD = −5.01%, *p* = 0.172) (Figure 4). Heterogeneity was estimated as extremely high (*I* ^2 ^= 97%). No publication bias was (*p* = 0.948, Egger´s test).

For cell therapy, only one study^42^ reported histologic outcomes. At 6 weeks, micro computerized tomography (micro‐CT) demonstrated a trend towards increased bone volume fraction and bone mineral density for sites treated with cell therapy versus GBR. Histomorphometric analyses demonstrated a higher bone area/tissue area at sites receiving cell‐therapy when compared to GBR. These differences were not observed in samples collected at the 12‐week re‐entry time point.

#### 3.2.3 Adverse events and complications

For biologics, three studies^25^, ^32^, ^35^ reported minor complications that did not differ between groups. Erythema was the most frequent post‐operative complication. No major adverse events were reported.

For cell‐therapy, data from one study^42^ demonstrated no serious adverse events for cell‐therapy or GBR.

#### 3.2.4 Patient‐reported outcome measures

For biologics, PROs were reported in two studies.^24^, ^25^ In general, post‐operative discomfort as measured by visual analog scale (VAS) was lower for test compared to control groups. Moreover, patient‐reported willingness to repeat the intervention was high for individuals who received treatments with the use of biologics.

For cell‐therapy, PROs were not reported.

#### 3.2.5 Quality assessment

Quality assessment for each of the included studies was based on the RoB 2 tool for assessing the risk of bias for RCTs. Most of the included studies revealed mid to low risk of bias (Supplementary Figure S1). Studies with high risk of bias were, in part, due to the lack of a reported description of the randomization process.

### 3.3 Maxillary sinus floor elevation

#### 3.3.1 Dimensional changes

**FIGURE 5:**
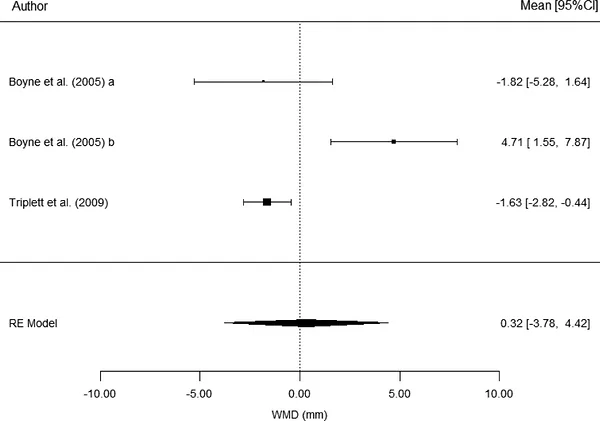
Meta‐analysis showing weighted mean differences (WMD) for the outcomes ridge height for sinus floor elevation.

For biologics, based upon two studies,^20^, ^74^ meta‐analysis demonstrated a WMD = 0.32 mm suggesting a higher gain of RH for the test groups compared to control interventions. Statistical significance, however, was not reached (*p* = 0.878) (Figure 5). Heterogeneity was high for this analysis (*I* ^2 ^= 87%). The following Funnel plot showed overall symmetry (*p* = 0.672, Egger's test).

For cell‐therapy, four studies^41^, ^62^, ^64^, ^80^ reported on dimensional changes. No differences were observed between cell‐therapy with scaffolds compared to control groups.

#### 3.3.2 Histologic outcomes

**FIGURE 6:**
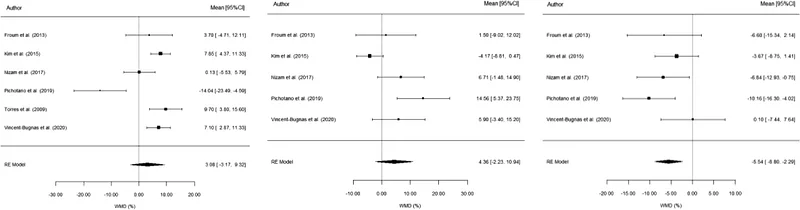
Meta‐analysis showing weighted mean differences (WMD) for the histologic outcomes % mineralized tissue (left panel), % non‐mineralized tissue (middle panel), and % residual graft (right panel) for sinus floor elevation.

For biologics, based on six studies,^34^, ^47^, ^58^, ^60^, ^73^, ^75^ meta‐analysis demonstrated a WMD = 3.08%, favoring biologics in terms of mineralized tissue. Nevertheless, statistical significance was not reached (*p* = 0.334). Heterogeneity was estimated as very high (*I* ^2 ^= 87%). Moreover, publication bias was detected (*p* = 0.022, Egger's test). Likewise, no differences were achieved in terms of percentage of non‐mineralized tissue^34^, ^47^, ^58^, ^60^, ^75^ (*p* = 0.19) with a moderate–high heterogeneity (*I* ^2 ^= 70%). Conversely, the percentage of residual graft was significantly lower for test groups (WMD = −5.54, *p* < 0.001) (Figure 6).^34^, ^47^, ^58^, ^60^, ^75^ Heterogeneity was estimated low (*I* ^2 ^= 21%).

For cell‐therapy, three studies^31^, ^61^, ^64^ reported on histologic outcomes. No significant differences were noticed in terms of mineralized tissue or residual particles. However, one study^31^ demonstrated higher percentage of mature bone for sites treated with cell‐therapy compared to maxillary sinus floor elevation using bone substitute alone.

#### 3.3.3 Adverse events and complications

For biologics, the use of rhBMP‐2 was associated with increased extra‐ and intra‐oral edema and prolonged post‐operative swelling. Nevertheless, post‐operative pain and rash were substantially less common with the use of rhBMP‐2.^20^ No major adverse events were noted.

For cell‐therapy, two study^64^, ^80^ suggested no adverse events or complications for cell‐therapy in maxillary sinus floor elevation

#### 3.3.4 Patient‐reported outcome measures

For biologics, the use of PRP was associated with an increased willingness to undergo the procedures again.^52^

For cell‐therapy, PROs reported in one study^61^ indicated patient satisfaction.

#### 3.3.5 Quality assessment

Quality assessment for each of the included studies was based on the RoB 2 tool for assessing the risk of bias in randomized controlled trials (RCTs). For the included non‐randomized studies, the ROBINS‐I v2 scale was used. Four studies^23^, ^50^, ^74^, ^75^ were scored to be at high risk of bias, primarily due to the lack of a detailed description of the randomization process in their methodologies. For non‐randomized studies, one study^54^ was assessed to have a high risk of bias due to substantial baseline imbalances between groups, particularly in residual bone height, a variable strongly associated with treatment outcomes

### 3.4 Implant‐related outcomes

#### 3.4.1 Implant stability and peri‐implant tissue stability

For biologics, while implant‐related outcomes were outside of the scope of the quantitative analysis in this report, it was noted that marginal bone loss reported on periapical x‐rays demonstrated similar outcomes to implants placed in simultaneously grafted sites with and without the use of adjunctive biologics.^29^, ^40^ Implant stability quotient (ISQ) was comparable between test and control groups during implant placement and at 18‐month follow‐up.^29^

For cell‐therapy, no studies met the inclusion criteria.

#### 3.4.2 Histologic outcomes

Applicable histologic outcomes were not reported after dental implant placement and could not be analyzed for biologics nor cell‐therapy

#### 3.4.3 Adverse events and complications

For biologics, minor differences were observed concerning the degree of post‐operative swelling between test and control groups.^40^ For cell‐therapy, no studies met the inclusion criteria.

#### 3.4.4 Patient‐reported outcome measures

For biologics, post‐operative discomfort as measured by VAS was comparable between groups.^40^ For cell‐therapy, no studies met the inclusion criteria.

#### 3.4.5 Quality assessment

Quality assessment for each of the included studies was based on the RoB 2 tool for assessing the risk of bias in RCTs. For the included non‐randomized studies, the ROBINS‐I v2 scale was used. One study^40^ was scored as a high risk of bias due to the lack of a reported description of the randomization process. Both non‐randomized studies presented high risk of bias, particularly due to lack of adjustment for anatomical and patient‐level prognostic variables. One study^15^ utilized a within‐patient control design aimed at reducing some variability, but such methodology cannot fully control for site‐specific confounders. In another study,^28^ consecutive recruitment was performed in two private clinics; however, the inclusion criteria may have preferentially selected patients with more favorable prognostic profiles, thereby increasing the risk of selection bias.

## 4 DISCUSSION

### 4.1 Summary of main findings

Alveolar bone regeneration is required in many clinical scenarios in contemporary implant dentistry to provide appropriate bone volume to stabilize dental implants in optimal positions to allow long‐term esthetics and function free of biological complications. The clinical challenges in achieving ideal outcomes after these ISD procedures is often complicated by advanced bone loss, ineffective bone metabolism, and altered bone microarchitecture related to patient‐related factors including systemic health, nutritional habits, deleterious habits such as smoking, age, or medication, among others. For these reasons, the use of biologics and cell‐therapy have been advocated to overcome the aforementioned limitations. This systematic review aimed at assessing the existing evidence on the adjunctive use of biologics and cell‐therapy for ISD interventions considering the very specific (focusing on specific interventions), relatively out to date and/or reviews that limited to qualitative assessment reported in the literature and published elsewhere.^1^, ^3^, ^4^, ^5^, ^6^, ^7^, ^8^ The use of biologics and cell‐therapy have been found to be safe and effective, with no adverse events or major complications associated with the use of adjunctive biologic mediators. Furthermore, PROs were, in general, minimally or not impacted by the use of the tested therapeutic strategies. Based upon the current data, meta‐analysis was only feasible for the use of biologics but not cell therapies. Interestingly, for ridge augmentation, the use of biologics resulted in superior outcomes in RW gain, especially the use of PRP (*p* < 0.001), but no differences were seen in RH with the use of biologics. For alveolar ridge preservation procedures, adjunctive use of biologics outperformed ridge preservation procedures alone in terms of RW and RH gain. The use of bone morphogenetic protein‐2 appeared to be particularly impactful for RW (*p* = 0.02) and RH (*p* = 0.09). For ridge preservation procedures, histologic analysis demonstrated less non‐mineralized tissue when adjunctive biologics were used compared to control groups (*p* = 0.009). For maxillary sinus floor elevation, no benefit was shown in terms of RH gain (*p* = 0.87). However, statistically less residual graft material was noticed when biologics were used in combination to scaffolds compared to scaffolds alone (*p* < 0.001). All in all, it was demonstrated that the use of biologics is beneficial in ridge augmentation and alveolar ridge preservation procedures. However, their impact in maxillary sinus floor elevation may be marginally beneficial in histologic but not in dimensional outcomes. Regardless the favorable findings showed in the present review regarding the use of biologics for ISD, cautiousness is suggested in interpreting these findings given the wide array of biologics explored, the different scaffold used, and the analytical methods applied that pooled different interventions/ comparisons together due to the heterogeneity reported in the included studies. Further, readers should review the individual clinical scenarios in each included study to account for the potential site‐specific impact on regenerative outcomes.

### 4.2 Agreements and disagreements with existing literature

Our findings partially agree, therefore, with findings reported in 2022 from the Best Evidence Consensus (BEC) commissioned by the American Academy of Periodontology (AAP) that aimed at analyzing the effect of biologics on the outcomes of ISD with or without scaffolds.^4^ Applying a relatively similar inclusion criteria, the number of publications included in the current review was significantly higher. As consequence, meta‐analysis could be performed in contrast to the BEC systematic review that reported only the findings form the qualitative analysis. Interestingly, while, overall, we reported that biologics combined with scaffolds for ridge augmentation/preservation procedures may elicit superior outcomes in terms of dimensional gain and histological new bone formation and mineralization, the review published at the BEC concluded that biologics in combination with a bone graft material generally result in superior histomorphometric outcomes and faster wound healing compared with control groups. Nonetheless, data derived from the previously performed qualitative assessment did not yield superior clinical outcomes for alveolar ridge preservation/augmentation in ISD procedures.^4^ It should also be noted that, similar to the 2022 AAP BEC, these analyses eliminated studies that included less than 10 subjects per arm. This common exclusion in both reviews should be noted, and clinicians and researchers should be aware of specific smaller‐scale studies that may be applicable to individual clinical situations that are not included in these analyses. The clinical findings reported in this review aligned with expert recommendations from the 2022 AAP BEC and may reflect the enhanced investigation of clinical outcomes related to biologics in combination with scaffolds following the 2022 AAP BEC and publication of their findings.^4^

### 4.3 In‐depth assessment of the findings

**FIGURE 7:**
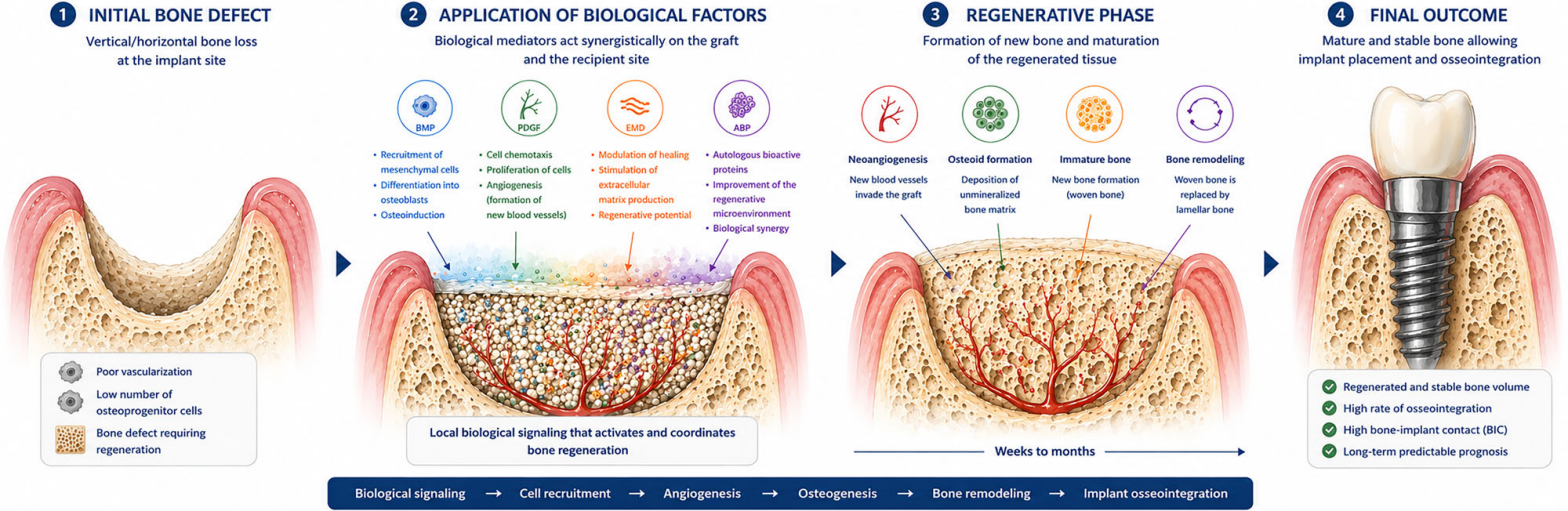
Mechanism of action of the different biological mediators.

During ISD hard‐tissue development procedures, biomaterials are meant to support alveolar bone regeneration process through maintaining volumetric stability and provision of a scaffold at the defect while undergoing resorption in situ and promoting replacement with newly formed bone (Figure 7). The selection of biomaterials is often dictated by the type of defect present and/or the need to sustain space maintenance and volumetric stability over extended time periods to account for longer healing processes. For instance, xenografts combined with autogenous bone have been suggested for use in guided bone regeneration aimed at lateral/vertical ridge augmentation. The rationale behind this combination relies on the physical and biological properties supplied by this mixture of bone fillers.^81^ In fact, demineralized bovine bone mineral was the most frequently tested material for test and control groups evaluating the effectiveness of this procedure.^14^, ^37^, ^39^, ^70^, ^72^, ^78^ In clinical practice, a variety of materials may be used with acceptable results, including allografts^30^ or bone blocks.^63^, ^70^ Collectively, the use of resorbable barrier membranes was common for guided bone regeneration.^14^, ^27^, ^30^, ^37^, ^39^, ^70^ Guided bone regeneration is based upon the principle of compartmentalization. Compartmentalization allows for the creation of different compartments to favor the exclusion of fibroblasts and epithelial cells from areas where the objective of the procedure to result in newly formed bone. Membranes have also been suggested to provide containment and support to the bone filler materials. In the case of ridge augmentation, the use of autologous blood‐derived growth factors (i.e., platelet rich plasma/fibrin) or the use of PRP/PRF in combination with bone grafting material may result in a more stable scaffold. It has been speculated that this mixture could enhance the healing and reconstructive outcomes thus, minimizing the use of collagen membranes.^72^, ^78^ In point of fact, one of the most commonly reported complications of the use of barrier membranes is their exposure and subsequent infection, although such complications are more common and potentially impactful on healing outcomes with the use of non‐resorbable membranes and barriers compared to resorbable membranes. One study^72^ demonstrated that the use of PRP may prevent titanium‐mesh exposure and that this strategy was linked with less risk for wound dehiscence. Decreased exposure was subsequently associated with significantly better outcomes in terms of vertical and lateral bone gain.

Allografts, on the other hand, were the most common scaffold used for ridge preservation.^17^, ^18^, ^24^, ^65^, ^66^ In scenarios where the extraction socket is intact and exhibits thick bony phenotype, the benefits of scaffold use may be negligible.^82^ Conversely, in extraction sockets characterized by thin bony phenotype in the anterior maxilla, the use of bone grafting materials simultaneous to tooth extraction or to implant placement seem to be critical to minimize volumetric changes during the healing period.^83^ The rationale for allograft use in extraction socket defects highlights that extraction site defects tend to demand less support due to their contained configuration.^84^ Moreover, the application of biologics for ridge preservation may result in enhanced soft tissue outcomes, particularly in open‐healing approaches, where primary closure is not achieved at the time of graft placement. Enhanced soft tissue closure may further positively impact biomaterial turnover used for ridge preservation. This may explain the increased non‐mineralized tissue presence upon histologic analysis for the use of biologics when compared to control groups (*p* = 0.009). It should also be noted that some of the included studies employed an early (8–12 week) reentry time frame for histologic sampling, therefore higher percentages of non‐mineralized tissues at sites where adjunctive biologics were used could reflect enhanced turnover and initial new bone formation at early time points which may not be discernable at later time points. Most of the biologics were tested for this intervention, with autologous blood‐derived growth factors being the most commonly used.^16^, ^17^, ^18^, ^24^, ^35^, ^51^, ^65^, ^66^ In addition, rhBMP‐2 were evaluated for this intervention.^25^, ^32^, ^38^, ^48^ In general, rhBMP‐2 was used with collagen sponges, since this is the suggested carrier for its on‐label use. Only one study^38^ used a combination of rhBMP‐2 with β‐tricalcium phosphate/hydroxyapatite. The use of rhBMP‐2 demonstrated superior dimensional outcomes RW (*p* = 0.02) and RH (*p* = 0.09) compared to control groups. This is not surprising since rhBMP‐2 are multi‐functional growth factors belonging to the transforming growth factor (TGF‐β) superfamily and activate osteogenic genes, such as *RUNX2* that induce rapid bone formation.^85^ One of the major shortcomings associated to the use of this biologic is increased soft tissue swelling that may compromise wound closure during the initial healing process.^86^ Reports have also demonstrated ectopic bone formation and seroma formation when rhBMP‐2 has been used, particularly at high dosages.^87^, ^88^ On a cellular level, this agent is known to be chemotactic for lymphocytes, monocytes, and macrophages.^89^ However, it was noticed from the included studies that no major early complications or adverse events were linked to the use of rhBMP‐2 in ridge preservation. Additionally, one study^53^ evaluated the effectiveness of demineralized bovine bone mineral with 10% collagen alone or with EMD in ridge preservation. No differences were noted in terms of dimensional ridge preservation outcomes; nevertheless, the addition of EMD to a scaffold resulted in superior outcomes in terms of newly formed bone at 4‐month re‐entry. Another study^42^ explored to safety and feasibility of stem cell‐therapy isolated from bone marrow and enriched in CD90‐ and CD14‐positive cells. Strikingly, this strategy, compared to conventional guided bone regeneration reduced significantly the need for secondary bone grafting at the time of oral implant placement with a 5‐fold decrease in implant bony dehiscence exposure (residual bone defects). More recently, a multicenter RCT demonstrated statistically significant superiority in bone volume for the performance of guided bone regeneration medicated by cell‐therapy when compared to autologous blocks.^79^ These findings demonstrated the effectiveness of this strategy; however, due to the limited data in this context, cautiousness must be advised concerning their use due to risks reported in the medical literature such as toxicity, neoplasm formation or undesired immune reaction.^90^

Maxillary sinus floor elevation procedures present defects that are, for the most part, completely contained and allow for predictable new bone formation with a variety of bone graft materials.^91^ A key factor in successful enhancement of RH during maxillary sinus floor elevation procedures is volumetric stability and the tenting of the Schneiderian membrane during the bone healing phase. Due to its minimal resorption and turnover over time, xenograft materials have been proposed as an ideal component of particulate graft materials used for maxillary sinus floor elevation. In clinical practice and published reports, the most common and predictable materials are xenograft and/or xenograft in combination with autograft or allograft particulate grafts. Further, maxillary sinus floor elevation may be accomplished through lateral window or crestal approaches and may or may not include simultaneous dental implant placement. The studies included in this analysis employed a variety of approaches and scaffold materials with demineralized bovine bone mineral being the most common. Given the excellent volumetric stability of this bone substitute and the relative containment of sinus defects, the finding that RH was not significantly influenced by the addition of adjunctive biologics was unsurprising. Further, several studies utilized either no carriers or collagen sponge carriers which provide little volumetric stability over time.^5^, ^23^, ^32^, ^53^ In these studies, the test group included biologics with no carrier or a collagen sponge which was then compared to standard mineralized particulate graft material.^5^, ^23^, ^32^, ^53^ These heterogeneous scaffold comparisons may have led to an obfuscation of the true impact of adjunctive biologics on RH in maxillary sinus floor elevation procedures within the meta‐analysis. It should be noted that the use of biologics was associated with decreased histologic residual graft particle presence, which could indicate faster graft resorption and turnover and indicate the capacity for earlier re‐entry and dental implant placement at sites where adjunctive biologics have been used in combination with particulate scaffolds. Among the included studies, healing times at assessment varied from 3 to 18 months, which may have also led to the equivocal findings related to histomorphometric impact of adjunctive biologics. Clinically, patients also seemed to prefer outcomes when biologics were used, with decreased pain and rash reported with rhBMP‐2 use and an increased willingness to undergo the procedure again reported with PRP^25^, ^53^ and no major adverse events were noted. Such findings may also be related to an accelerated healing timeline associated with the use of biologics. While these findings should be taken with caution, they do suggest that the addition of adjunctive biologics could increase bone turnover and new bone formation at earlier time points and lead to enhanced protocols for the timing of implant placement and/or restoration.

While quantitative analysis was not feasible to evaluate the impact of adjunctive biologic use during simultaneous grafting with implant placement, no significant impact of the use of biologics on marginal bone levels were identified at up to 60 months following the procedures.^35^, ^44^ Similarly, no differences were seen in implant ISQ values at 18 months after placement.^44^ It should also be noted that in one of the included studies PRF without mineralized scaffold was compared to the use of DBBM and alloplastic mineralized particulate graft use,^44^ which may provide clinicians with flexibility when choosing materials for onlay grafting of implant defects based upon defect morphology, patient preference, and other concerns. While these findings do not indicate a rationale for the use of adjunctive biologics during grafting for implant placement at fresh extraction or healed extraction sites, it is unknown if earlier measurements of ISQ would have indicated differences in healing trajectories between groups. Further, because tissue assessments in these investigations were radiographic in nature, only the hard tissue could be quantified and the impact of adjunctive biologics on soft tissue quality and quantity is unknown.

While evidence is limited to support the use of cell‐therapy in guided bone regeneration applications, data from some initial studies^31^, ^42^ may indicate that the use of cell‐therapy may improve initial healing and histologic maturation of hard tissues. This could potentially reduce healing and treatment time for patients seeking implant therapy for tooth replacement at sites with deficient bone. It was also noted that the need for simultaneous grafting was reduced at sites where cell‐therapy was used. While much is still unknown regarding ideal scaffolds, the impact of pluripotent stem cells on downstream healing events, and optimal clinical indications for stem cell use, future research into these areas could result in enhanced understanding of the contributions of cellular components to healing and more thoroughly define ideal clinical protocols.

### 4.4 Limitations and potential biases

Despite the adequate number of manuscripts identified though the systematic screening, fewer met the criteria for meta‐analysis. For this reason, findings from this review paper should be interpreted with caution. It is relevant to bear in mind that non‐randomized trials and comparative studies were included to increase sample size and, thus, the weight of the findings. However, the quality and consistency of the outcomes might be partly sacrificed by the inclusion of these data. Moreover, some trials reported site‐level outcomes without adjusting for within‐patient clustering, while others used patient‐level summaries. As clustering adjustments were not possible with the available data, results should be interpreted in light of these methodological constraints. Moreover, it is important to highlight that the clinical applications of biologics were inconsistent regarding type of biologic and surgical interventions, scaffold choice, timing of application, and dosage. For instance, different properties have been suggested according to the centrifugation method/time for various autologous blood‐derived growth factors.^92^ Limited information was provided on the specific biologic preparation protocols, which may limit the generalizability of these findings. Additionally, biologics may perform differently based upon the type of scaffold utilized. However, given the high heterogeneity among the included reports, subset meta‐analyses based upon biologic and scaffold combinations were not feasible. While it has been proposed that adjunctive use of biologics may be advantageous in individual patient who present with systemic conditions impacting wound healing and immune function, such as those with diabetes mellitus and autoimmune dysfunction,^1^ based upon the inclusion criteria in this review, all evaluated patients were systemically healthy. Clinicians must, therefore, seek out additional literature and/or utilize their clinical judgement when assessing the utility of biologics and cell therapies in individuals with systemic conditions that may impact surgical outcomes of alveolar bone augmentation for ISD. It should also be noted that meta‐analyses may result in a regression to the mean when some subgroups demonstrate large intervention effect size and others demonstrate minimal or no effect.^93^ This highlights the need for further research and consistent protocols to allow for larger‐scale analyses with broader clinical applications. This review and meta‐analysis (where applicable) are meant to provide an initial qualitative and quantitative assessment of the current state‐of‐the‐art practices for the use of biologics combined with scaffolds for alveolar bone augmentation. To allow enhanced data interpretation and subgroup analysis, it is suggested that future research adhere to ID‐COSM to report findings from studies using biologics or cell‐therapy for bone augmentation procedures aiming at ISD.

## 5 CONCLUSIONS

Within the limitations identified across the included studies, including substantial clinical and methodological heterogeneity, the following conclusions can be drawn:

- Biologics combined with scaffolds for ridge augmentation and alveolar ridge preservation procedures are suggested to be safe and to elicit superior outcomes in terms of dimensional gain and histological resemblance to native bone when compared to control groups.
- Qualitative analysis may suggest patient preferences for the use of biologics, particularly autologous blood‐derived products, to reduce post‐operative discomfort and sequelae.
- While no differences were reached in clinical and radiographic findings associated with the use of biologics during maxillary sinus floor elevation, an increase in the proportion of non‐mineralized tissues may indicate alterations in bone turnover and healing when adjunctive biologics are employed.
- The use of cell‐therapy seems to be safe and effective but due to the limited data currently published, its superiority to traditional methods for alveolar bone augmentation could not be validated.
- Cautiousness is suggested in interpreting these findings due to the high heterogeneity in terms of variables encountered in the included studies.
- Further research with standardized protocols, whenever possible with proper randomization and under equal clinical scenarios, could improve clarity and data interpretation for clinicians and dental researchers.

## AUTHOR CONTRIBUTIONS

**Alberto Monje**: Conceptualization; writing–original draft. **Maria Costanza Soldini**: Data curation; formal analysis. **Farahnaz Fahimipour**: Data curation; formal analysis. **Manal Sarsri**: Data curation; formal analysis. **Maria L. Geisinger**: Conceptualization; writing–original draft.

## CONFLICT OF INTEREST STATEMENT

The authors declare no conflicts of interest.

## Supporting information



Supporting Information

Supporting Information

Supporting Information

## Footnotes

* Any p‐value below 0.05 is indicative of a relation that is statistically significant. To the contrary, a p‐value above or equal to 0.05 indicates lack of relation.
