# Supplementary material for: Use of cells and/or biologics combined with scaffolds in alveolar bone augmentation for implant site development
Source: J Periodontol. 2026 Sep 14;97(Suppl 1):S70–88. doi: 10.1002/jper.70105 (PMC13575443; doi:10.1002/jper.70105)
Supplement: Supplementary file 1 [file JPER-97-S70-s001.docx]

**Supplementary figure 1. Risk of bias for studies of Ridge augmentation**

| **1** | **2** | **3** | **4** | **5** | **Overall** | **Study** |
| --- | --- | --- | --- | --- | --- | --- |
| 🟡 | 🟢 | 🟢 | 🟡 | 🟢 | 🟡 | Aboelela et al. (2022) |
| 🟢 | 🟡 | 🟢 | 🟡 | 🟢 | 🟡 | de Freitas et al. (2013) |
| 🟢 | 🟡 | 🟢 | 🟡 | 🟢 | 🟡 | Eskan et al. (2014) |
| 🟡 | 🟢 | 🟡 | 🟢 | 🟡 | 🟡 | Hartlev et al. (2021) |
| 🟢 | 🟢 | 🟢 | 🟡 | 🟡 | 🟡 | Isik al. (2021) |
| 🟢 | 🟢 | 🟢 | 🟡 | 🟡 | 🟡 | Nam et al. (2017) |
| 🟡 | 🟡 | 🟢 | 🟡 | 🟡 | 🟡 | Santana et al. (2015) |
| 🟢 | 🟢 | 🟢 | 🟢 | 🟢 | 🟢 | Sanz et al. (2025) |
| 🟡 | 🟢 | 🟡 | 🟡 | 🟡 | 🟡 | Thoma et al. (2018) |
| 🟡 | 🟡 | 🟢 | 🟡 | 🟡 | 🟡 | Thor et al. (2005) |
| 🟢 | 🟡 | 🟢 | 🟡 | 🟢 | 🟡 | Torres et al. (2010) |
| 🟢 | 🟢 | 🟢 | 🟢 | 🟢 | 🟢 | Xie et al. (2024) |

Footone:

1 = Bias arising from the randomisation process *(allocation sequence generation and concealment)*
2 = Bias due to deviations from intended interventions *(performance bias, adherence to intended interventions)*
3 = Bias due to missing outcome data *(attrition bias)*
4 = Bias in measurement of the outcome *(detection bias)*
5 = Bias in selection of the reported result *(reporting bias)*
Overall = Overall risk of bias across domains

🟢= low risk

🟡=moderate risk

🔴=high risk

**Fig 2. Risk of bias for studies of Ridge preservation**

| **1** | **2** | **3** | **4** | **5** | **Overall** | **Study** |
| --- | --- | --- | --- | --- | --- | --- |
| 🟢 | 🟢 | 🟢 | 🟢 | 🟢 | 🟢 | Abaza et al. (2023) |
| 🟢 | 🟢 | 🟡 | 🟡 | 🟡 | 🟡 | Areewong et al. (2019) |
| 🟡 | 🟡 | 🟡 | 🟡 | 🟡 | 🟡 | Alzahrani et al. (2017) |
| 🟢 | 🟢 | 🟢 | 🟢 | 🟢 | 🟡 | Azangookhiavi et al. (2024) |
| 🟢 | 🟢 | 🟢 | 🟢 | 🟡 | 🟡 | Canellas et al. (2020) |
| 🟢 | 🟢 | 🟢 | 🟢 | 🟢 | 🟢 | Castro et al. (2021) |
| 🟢 | 🟢 | 🟢 | 🟢 | 🟢 | 🟢 | Clark et al. (2018) |
| 🟡 | 🟢 | 🟢 | 🟢 | 🟢 | 🟡 | Coomes et al. (2014) |
| 🔴 | 🟢 | 🟢 | 🟢 | 🟢 | 🔴 | Fiorellini et al. (2005) |
| 🟢 | 🟢 | 🟢 | 🟢 | 🟢 | 🟢 | Han et al. (2011) |
| 🟢 | 🟢 | 🟢 | 🟢 | 🟡 | 🟡 | Kaigler al. (2013) |
| 🟢 | 🟢 | 🟢 | 🟢 | 🟡 | 🟡 | Kim et al. (2014) |
| 🔴 | 🟡 | 🟡 | 🟡 | 🟡 | 🔴 | Kumar et al. (2018) |
| 🟢 | 🟢 | 🟢 | 🟢 | 🟡 | 🟡 | Lahham et al. (2023) |
| 🟡 | 🟢 | 🟡 | 🟢 | 🟢 | 🟡 | Lin et al. (2021) |
| 🟡 | 🟢 | 🟢 | 🟢 | 🟢 | 🟡 | Mercado et al. (2021) |
| 🟡 | 🟢 | 🟢 | 🟢 | 🟢 | 🟡 | Mousavi et al. (2024) |
| 🟢 | 🟢 | 🟢 | 🟢 | 🟡 | 🟡 | Stumbras et al. (2020) |
| 🟢 | 🟢 | 🟢 | 🟢 | 🟡 | 🟡 | Stumbras et al. (2021) |
| 🟢 | 🟢 | 🟢 | 🟢 | 🟡 | 🟡 | Temmerman et al. (2016) |
| 🟢 | 🟢 | 🟢 | 🟢 | 🟢 | 🟢 | Wang et al. (2022) |

Footone:

1 = Bias arising from the randomisation process *(allocation sequence generation and concealment)*
2 = Bias due to deviations from intended interventions *(performance bias, adherence to intended interventions)*
3 = Bias due to missing outcome data *(attrition bias)*
4 = Bias in measurement of the outcome *(detection bias)*
5 = Bias in selection of the reported result *(reporting bias)*
Overall = Overall risk of bias across domains

🟢= low risk

🟡=moderate risk

🔴=high risk

**Fig 3. Risk of bias for studies of Maxillary Sinus lift (RCT)**

| **1** | **2** | **3** | **4** | **5** | **Overall** | **Study** |
| --- | --- | --- | --- | --- | --- | --- |
| 🟡 | 🟡 | 🟡 | 🟡 | 🟡 | 🟡 | Barros-Mourão CF et al. (2019) |
| 🟢 | 🟢 | 🟢 | 🟢 | 🟡 | 🟡 | Boyne et al. (2005) |
| 🔴 | 🟢 | 🟢 | 🟢 | 🟢 | 🔴 | Cho et al. (2020) |
| 🟢 | 🟢 | 🟢 | 🟢 | 🟢 | 🟢 | Froum et al. (2013) |
| 🟢 | 🟢 | 🟢 | 🟡 | 🟡 | 🟡 | Froum et al. (2013) |
| 🟢 | 🟢 | 🟢 | 🟢 | 🟡 | 🟡 | Kaigler et al. (2015) |
| 🟢 | 🟢 | 🟢 | 🟡 | 🟡 | 🟡 | Karaagh et al. (2022) |
| 🟢 | 🟢 | 🟢 | 🟡 | 🟡 | 🟡 | Khairy et al. (2013) |
| 🟢 | 🟢 | 🟢 | 🟡 | 🟡 | 🟡 | Kim et al. (2015) |
| 🔴 | 🟡 | 🟡 | 🟡 | 🟡 | 🔴 | Lee et al. (2009) |
| 🟢 | 🟢 | 🟢 | 🟡 | 🟡 | 🟡 | Lv et al. (2022) |
| 🟢 | 🟢 | 🟢 | 🟡 | 🟡 | 🟡 | Nizam et al. (2017) |
| 🟢 | 🟢 | 🟢 | 🟢 | 🟢 | 🟢 | Ozbay et al. (2024) |
| 🟢 | 🟢 | 🟢 | 🟡 | 🟡 | 🟡 | Pichotano et al. (2019) |
| 🟢 | 🟢 | 🟢 | 🟡 | 🟡 | 🟡 | Rickert et al. (2014) |
| 🔴 | 🟡 | 🟡 | 🟡 | 🟡 | 🔴 | Triplett et al. (2009) |
| 🟢 | 🟢 | 🟢 | 🟡 | 🟡 | 🟡 | Torres et al. (2009) |
| 🔴 | 🟢 | 🟢 | 🟡 | 🟡 | 🔴 | Vincent-Bugnas et al. (2020) |
| 🟡 | 🟡 | 🟡 | 🟡 | 🟡 | 🟡 | Wilfang et al. (2003) |

Footone:

1 = Bias arising from the randomisation process *(allocation sequence generation and concealment)*
2 = Bias due to deviations from intended interventions *(performance bias, adherence to intended interventions)*
3 = Bias due to missing outcome data *(attrition bias)*
4 = Bias in measurement of the outcome *(detection bias)*
5 = Bias in selection of the reported result *(reporting bias)*
Overall = Overall risk of bias across domains

🟢= low risk

🟡=moderate risk

🔴=high risk

**Fig 3.2 Risk of bias for studies of Maxillary Sinus lift (Non-RCT)**

| **1** | **2** | **3** | **4** | **5** | **6** | **7** | **Overall** | **Study** |
| --- | --- | --- | --- | --- | --- | --- | --- | --- |
| 🟡 | 🟢 | 🟢 | 🟢 | 🟡 | 🟢 | 🟢 | 🟡 | Bettega et al. (2009) |
| 🔴 | 🟡 | 🟢 | 🟢 | 🟢 | 🟡 | 🟡 | 🔴 | Merli et al. (2022) |
| 🟡 | 🟢 | 🟢 | 🟢 | 🟢 | 🟢 | 🟡 | 🟡 | Taschieri et al. (2016) |
| 🟡 | 🔴 | 🟢 | 🟡 | 🟡 | 🟡 | 🟡 | 🔴 | Tzur et al. (2021) |
| 🟡 | 🟢 | 🟢 | 🟢 | 🟢 | 🟢 | 🟡 | 🟡 | Testori et al. (2019) |

Footone:

1 = Bias due to confounding
2 = Bias in selection of participants into the study
3 = Bias in classification of interventions
4 = Bias due to deviations from intended interventions
5 = Bias due to missing data
6 = Bias in measurement of outcomes
7 = Bias in selection of the reported result
Overall = Overall risk of bias across domains

🟢= low risk

🟡=moderate risk

🔴=serious risk

🟥 =critical risk

**Fig 4.1 Risk of bias for studies of implants (RCT)**

| **1** | **2** | **3** | **4** | **5** | **Overall** | **Study** |
| --- | --- | --- | --- | --- | --- | --- |
| 🟢 | 🟢 | 🟢 | 🟢 | 🟡 | 🟡 | Darestani et al. (2023) |
| 🟢 | 🟢 | 🟢 | 🟢 | 🟢 | 🟢 | Elsheikh et al. (2023) |
| 🔴 | 🔴 | 🔴 | 🟢 | 🟡 | 🔴 | Jung et al. (2009) |

Footone:

1 = Bias arising from the randomisation process *(allocation sequence generation and concealment)*
2 = Bias due to deviations from intended interventions *(performance bias, adherence to intended interventions)*
3 = Bias due to missing outcome data *(attrition bias)*
4 = Bias in measurement of the outcome *(detection bias)*
5 = Bias in selection of the reported result *(reporting bias)*
Overall = Overall risk of bias across domains

🟢= low risk

🟡=moderate risk

🔴=high risk

**Fig 4.2 Risk of bias for studies of Implants (Non-RCT)**

| **1** | **2** | **3** | **4** | **5** | **6** | **7** | **Overall** | **Study** |
| --- | --- | --- | --- | --- | --- | --- | --- | --- |
| 🔴 | 🟡 | 🟢 | 🟢 | 🟢 | 🟡 | 🟡 | 🔴 | Al Nashar et al. (2015) |
| 🔴 | 🔴 | 🟢 | 🟢 | 🟢 | 🔴 | 🔴 | 🔴 | Del Fabbro et al. (2009) |

Footone:

1 = Bias due to confounding
2 = Bias in selection of participants into the study
3 = Bias in classification of interventions
4 = Bias due to deviations from intended interventions
5 = Bias due to missing data
6 = Bias in measurement of outcomes
7 = Bias in selection of the reported result
Overall = Overall risk of bias across domains

🟢= low risk

🟡=moderate risk

🔴=serious risk

🟥 =critical risk
