# Supplementary material for: Use of cells and/or biologics combined with scaffolds in alveolar bone augmentation for implant site development
Source: J Periodontol. 2026 Sep 14;97(Suppl 1):S70–88. doi: 10.1002/jper.70105 (PMC13575443; doi:10.1002/jper.70105)
Supplement: Supplementary file 2 [file JPER-97-S70-s002.docx]

# Supplementary table 1. Electronic search strategies specific to each database

| Database | Search Strategy | Filters/Restrictions Applied |
| --- | --- | --- |
| PubMed  N=3818 | (((((((((((((((((((((((((((edentulous jaw[MeSH Terms]) OR (edentulous mouth[MeSH Terms])) AND (edentulous alveolar ridge)) OR (alveolar ridge augmentation[MeSH Terms])) OR (mandibular ridge augmentation[MeSH Terms])) OR (maxillary ridge augmentation[MeSH Terms])) OR (maxillary sinus floor augmentation[MeSH Terms])) OR (sinus floor augmentation[MeSH Terms])) OR (sinus floor elevation[Title])) OR (alveolar ridge preservation[Title])) OR (socket preservation[Title])) OR (horizontal ridge augmentation[Title])) OR (horizontal bone augmentation[Title])) OR (vertical ridge augmentation[Title])) OR (sinus floor[Title])) AND (vertical bone augmentation[Title])) OR (platelet growth factor[Title/Abstract])) OR (enamel matrix derivative[Title/Abstract])) OR (platelet derived growth factor[Title/Abstract])) OR (EMD[Title/Abstract])) OR (Emdogain[Title/Abstract])) OR (PDGF[Title/Abstract])) OR (PRP[Title/Abstract])) OR (PPP[Title/Abstract])) OR (PRF[Title/Abstract])) OR (platelet rich fibrin[Title/Abstract])) AND (GEM-21[Title/Abstract])) AND (cell therapy[Title/Abstract])) OR (bone augmentation[Title/Abstract])) OR (bone gain[Title/Abstract])) OR (implant survival[Title/Abstract])) OR (bone loss[Title/Abstract]) | No date restrictions. Language: English. Human studies. |
| EMBASE  N = 248 | (  'edentulous jaw'/exp OR  'edentulous mouth'/exp OR  'edentulous ridge'/exp OR  'alveolar ridge augmentation'/exp OR  'mandibular ridge augmentation'/exp OR  'maxillary ridge augmentation'/exp OR  'maxillary sinus floor augmentation'/exp OR  'sinus floor augmentation'/exp OR  'sinus floor elevation'/exp OR  'alveolar ridge preservation'/exp OR  'socket preservation'/exp OR  'horizontal ridge augmentation'/exp OR  'horizontal bone augmentation'/exp OR  'vertical ridge augmentation'/exp OR  'vertical bone augmentation'/exp OR  'bone augmentation'/exp OR  'bone gain'/exp OR  'bone loss'/exp OR  'dental implant survival'/exp  )  AND  (  'platelet growth factor'/exp OR  'enamel matrix derivative'/exp OR  'platelet derived growth factor'/exp OR  'emd'/exp OR  'emdogain'/exp OR  'pdgf'/exp OR  'platelet rich plasma'/exp OR  'prp'/exp OR  'ppp'/exp OR  'platelet rich fibrin'/exp OR  'prf'/exp OR  'gem 21'/exp OR  'cell therapy'/exp  ) | Filter: Randomized Clinical Trials. Language: English.. |
| Cochrane Library  N=657 | (  "edentulous jaw":ti,ab,kw OR  "edentulous mouth":ti,ab,kw OR  "edentulous alveolar ridge":ti,ab,kw OR  "alveolar ridge augmentation":ti,ab,kw OR  "mandibular ridge augmentation":ti,ab,kw OR  "maxillary ridge augmentation":ti,ab,kw OR  "maxillary sinus floor augmentation":ti,ab,kw OR  "sinus floor augmentation":ti,ab,kw OR  "sinus floor elevation":ti,ab,kw OR  "alveolar ridge preservation":ti,ab,kw OR  "socket preservation":ti,ab,kw OR  "horizontal ridge augmentation":ti,ab,kw OR  "horizontal bone augmentation":ti,ab,kw OR  "vertical ridge augmentation":ti,ab,kw OR  "vertical bone augmentation":ti,ab,kw OR  "bone augmentation":ti,ab,kw OR  "bone gain":ti,ab,kw OR  "bone loss":ti,ab,kw OR  "dental implant survival":ti,ab,kw  )  AND  (  "platelet growth factor":ti,ab,kw OR  "enamel matrix derivative":ti,ab,kw OR  "platelet derived growth factor":ti,ab,kw OR  emd:ti,ab,kw OR  emdogain:ti,ab,kw OR  pdgf:ti,ab,kw OR  prp:ti,ab,kw OR  ppp:ti,ab,kw OR  prf:ti,ab,kw OR  "platelet rich fibrin":ti,ab,kw OR  "gem 21":ti,ab,kw OR  "cell therapy":ti,ab,kw  ) | Filter: Randomized Clinical Trials; Language: English |

*All searches were conducted up to and including 1 November 2024. The search results were imported into Rayyan software, and duplicate records were identified and removed using the program’s automated duplicate detection feature.*
