# Supplementary material for: Use of cells and/or biologics combined with scaffolds in alveolar bone augmentation for implant site development
Source: J Periodontol. 2026 Sep 14;97(Suppl 1):S70–88. doi: 10.1002/jper.70105 (PMC13575443; doi:10.1002/jper.70105)
Supplement: Supplementary file 3 [file JPER-97-S70-s003.docx]

**Table 1.** Included studies in the qualitative assessment.

| **Intervention** | **Author** | **Setting(s)** | **Country**  | **Approach** | **Study design** | **Biologic** | **Scaffold** | **Interventions per Study Group** | **Initial Number of Participants and  Group Distribution** | **Drop-outs at Different Follow-up Time Points** | **Final Number of Participants** | **Healing time at examination (months)** |  |  | **Dimensional outcomes** | | | | **Histologic outcomes** | | | | **Implant stability outcomes** | | **PROMs** | **Adverse effects** | **Conflict of Interest** |
| --- | --- | --- | --- | --- | --- | --- | --- | --- | --- | --- | --- | --- | --- | --- | --- | --- | --- | --- | --- | --- | --- | --- | --- | --- | --- | --- | --- |
|  |  |  |  |  |  |  |  |  |  |  |  |  | **Method of assessment** | **Primary objective** | **Ridge dimension  Baseline** Mean + SD | **Re-assessment  Final** Time (Months) | **Ridge dimension  Final** Mean + SD | **Ridge dimension  Change** Mean + SD | Density x-ray (%) | Mineralized tissue (%) | Non-mineralized tissue (%) | Residual graft (%) | ISQ value | Insertion torque |  |  |  |
| **Ridge augmentation** | **Aboelela et al. (2022)** | University; Single center | Egypt | Lateral ridge augmentation | RCT | CGF | Autogenous bone graft + DBBM particles | Augmentation with 1:1 mixture of particulate autogenous bone and DBBM autologous fibrin glue to form "sticky bone," covered by a concentrated growth factor membrane. | 14 | 0 | 14 | 6 | •CBCT •RFA | RW | 6.09 ± 0.83 mm | 6 | 7.90 ± 0.92 mm | NA | NA | NA | NA | NA | •Baseline: 66.74 (3.22)  •6m: 72.00 (2.15) | NA | NA | NA | The authors declare no conflict of interest. |
|  |  |  |  |  |  | _ | Autogenous bone graft + DBBM particles | Augmentation with 1:1 mixture of particulate autogenous bone and DBBM covered with a native collagen membrane. | 14 | 0 | 14 | 6 |  |  | 6.77 ± 0.80 mm | 6 | 9.00 ± 0.71 mm | NA | NA | NA | NA | NA | •Baseline 67.19 (2.23)  •6m:71.77 (2.27) | NA | NA | NA |  |
|  | **de Freitas et al. (2013)** | University; Single center | Brazil | Lateral ridge augmentation | RCT | rhBMP-2 | ACS | Augmentation of the anterior maxilla with rhBMP-2/ACS (1.5 mg/ml; 4.2 mg per site) in an absorbable collagen sponge carrier. | 12 | 0 | 12 | 6 | •Clinical measuraments (caliper) •CBCT | RW | •Radiographic: 3.6 ± 1.0 mm •Clinical: 2.1 ± 0.8 mm | 6 | NA | •Radiographic: 1.5 ± 0.7 mm  •Clinical: 3.2 ± 0.9 mm | NA | NA | NA | NA | NA | NA | NA | •Facial swelling, local oedema and/or erythema more frequent and severe among subjects receiving rhBMP-2/ACS | None reported |
|  |  |  |  |  |  | _ | Autogenous bone particles | Augmentation of the anterior maxilla with autogenous bone graft. | 12 | 0 | 12 | 6 |  |  | •Radiographic: 4.0 ± 0.7 mm •Clinical: 2.1 ± 0.8 mm | 6 | NA | •Radiographic: 0.5 ± 0.9 mm  •Clinical: 3.7 ± 1.4 mm | NA | NA | NA | NA | NA | NA | NA | •Temporary discomfort and/ or pain from the donor site |  |
|  | **Eskan et al. (2014)** | University; Single center | USA | Lateral ridge augmentation | RCT | PRP | Mineralized particulate cancellous allograft | Augmentation with PRP and cancellous allograft and covered with a resorbable polylactide membrane. | 16 | 2 | 14 | 4 | •Clinical measuraments (caliper) •Histology | RW | 3.4 ± 1.0 mm | 4 | 6.3 ± 1.5 mm | 2.9 ± 1.0 mm | NA | 51 ± 15 | NA | 6 ± 5 | NA | NA | NA | NA | The authors reported no conflicts of interest |
|  |  |  |  |  |  | _ | Mineralized particulate cancellous allograft | Augmentation with a cancellous allograft and covered with a resorbable polylactide membrane. | 16 | 2 | 14 | 4 |  |  | 3.5 ± 1.1 mm | 4 | 5.5 ± 1.4 mm | 2.0 ± 1.2 mm | NA | 36 ± 14 | NA | 10 ± 5 | NA | NA | NA | 0 |  |
|  | **Gjerde et al. (2018)** | University; Single center | Norway | Lateral ridge augmentation | Prospective clinical study | MSC | Biphasic calcium phosphate granules | Augmentation with MCS and iphasic calcium phosphate granules as scaffold. Titanium-reinforced, nonresorbable polytetrafluoroethylene membranes were then fixed to the underlying bone by micro-screws and mini-screws. | 13 | 2 | 11 | 6 | •CBCT •micro CT •Histology | RW | NA | NA | NA | 4.05 (95% CI 2.74, 5.36) mm | NA | NA | NA | NA | NA | NA | •One patient reported moderate levels of pain after augmentation and after the exposed membrane had to be removed. • The other patients reported only minor pain postoperatively.  •All patients were satisfied with the clinical outcome of the augmentation procedure and with their new teeth.  •All patients said they would recommend this procedure to others with a similar clinical condition. | 0 | Financial support of the European Union’s Seventh Framework Programme under grant agreement number 241879—REBORNE is acknowledged. The authors also acknowledge the significant contribution by Institut Straumann AG (Basel, Switzerland) for providing the dental implants, Osteogenics Biomedical (Lubbock, TX, USA for the Cytoplast polytetrafluoroethylene membranes, and Ostell (Gothenburg, Sweden) for the Ostell device. |
|  | **Hartlev et al. (2021)** | University; Single center | Denmark | Lateral ridge augmentation | RCT | PRF | Autogenous bone particles | Augmentation with autogenous bone block was covered by autogenous bone graft particles and a PRF membrane. Dental implant was placed after 6 months. | 13 | 0 | 13 | 24 | •RX •Questionnaire | NA | NA | NA | NA | NA | NA | NA | NA | NA | NA | NA | • VAS: The change from baseline to follow-up examination was 0.24 (95% CI: − 0.37 to 0.85, p = 0.43) units higher for the PRF group than the control group. | • One patient: sensory disturbances at the mucosa of the alveolar sulcus at the recipient site. | Geistlich Pharma AG (Geistlich Bio-Oss® Spongiosa Granules, Geistlich Bio- Gide®) and Nobel Biocare® (NobelParallel Conical Connection) provided the materials used in this study free of charge. |
|  |  |  |  |  |  | _ | DBBM particles | Augmentation with autogenous bone block was covered by DBBM bone graft particles and a resorbable collagen membrane. Dental implants were placed after 6 months. | 14 | 0 | 14 | 24 |  |  | NA | NA | NA | NA | NA | NA | NA | NA | NA | NA |  | • One patient: minimally changed extraoral sensation in the chin region at both the 1- and 2-week follow-up |  |
|  | **Isik al. (2021)** | University; Single center | Turkey | Lateral ridge augmentation | RCT | PRF | DBBM particles | Augmentation with PRF-enriched bovine-derived xenograft and a resorbable membrane. | 22 | 2 | 20 | 6 | •CBCT | RW | 4.25 ± 0.26 mm | 6 | NA | 1.63 ± 0.21 mm | NA | NA | NA | NA | NA | NA | NA | NA | No conflict of interest reported |
|  |  |  |  |  |  | _ | DBBM particles | Augmentation with bovine-derived xenograft and a resorbable membrane. | 22 | 2 | 20 | 6 |  |  | 4.33 ± 0.28 mm | 6 | NA | 1.34 ± 0.14 mm | NA | NA | NA | NA | NA | NA | NA | NA |  |
|  | **Khojasteh et al. (2016)** | University; Single center | Iran | Lateral ridge augmentation | CT | BFPSC | Autogenous bone blocks + FDBA | Augmentation with onlay bone block grafting from the anterior iliac crest. FDBA and collagen membranes were used to cover the defects. | 4 | 0 | 4 | 5 | •CBCT •Histology | RW | 1.96 ± 0.98 mm | 5 | 5.93 ± 1.28 mm | 3.01 ± 0.89 mm | NA | NA | NA | NA | NA | NA | NA | NA | No conflict of interest reported. This study was supported by the Dental Research Centre, Research Institute of Dental Sciences, Shahid Beheshti Univer- sity of Medical Sciences. |
|  |  |  |  |  |  | _ | Autogenous bone blocks + FDBA | Augmentation with onlay bone block grafting from the anterior iliac crest.FDBA granules were loaded with autogenous MSCs harvested from the buccal fat pad and collagen membranes were used to cover the defects. | 4 | 0 | 4 | 5 |  |  | 2.88 ± 1.12 mm | 5 | 5.84 ± 1.02 mm | 3.94 ± 1.62 mm | NA | NA | NA | NA | NA | NA | NA | NA |  |
|  | **Khojasteh et al. (2018)** | University; Single center | Iran | Vertical and lateral ridge augmentation | RCT | BFPSC | DBBM particles | Augmentation with xenograft particles and with autogenous MSCs harvested from the buccal fat pad. Titanium mesh was contoured to the desired 3D shape of the alveolar ridge and fixated to the host sites over the graft material. | 7 | 0 | 7 | 6 | •CBCT •Histology •SEM •Flowcytometry | RH; RW | NA | NA | NA | •RH: 164.91 ± 3.74 mm2  •RW: 170.51 ± 4.54 mm2 | NA | NA | NA | NA | NA | NA | NA | NA | No conflict of interest reported |
|  |  |  |  |  |  | _ | DBBM particles + autogenous bone particles | Augmentation with autogenous bone graft and xenograft particles. Titanium mesh was contoured to the desired 3D shape of the alveolar ridge and fixated to the host sites over the graft material | 7 | 0 | 7 | 6 |  |  | NA | NA | NA | •RH:169.36 ± 12.09 mm  •RW: 166.98 ± 9.36 mm2 | NA | NA | NA | NA | NA | NA | NA | NA |  |
|  | **Nam et al. (2017)** | University; Single center | Korea | Vertical ridge augmentation | RCT | rhBMP-2 | HA particles | Augmentation with HA particles . | 10 | 0 | 10 | 4 | •RX •Histology | RH | NA | 4 | NA | 7.12 ± 3.28 mm | NA | NA | NA | NA | NA | NA | NA | NA | No conflict of interest reported |
|  |  |  |  |  |  | _ | DBBM particles | Augmentation with small granule xenograft, | 10 | 0 | 10 | 4 |  |  | NA | 4 | NA | 7.31 ± 4.43 mm | NA | NA | NA | NA | NA | NA | NA | NA |  |
|  | **Santana et al. (2015)** | University; Single center | Brazil | Lateral ridge augmentation | RCT | rhPDGF-BB | β-TCP/HA | Augmentation with rhPDGF-BB incorporated into β-TCP/HA. | 15 | 0 | 15 | 6 | •Clinical measurements | RW | 3.13 ± 0.96 mm | 6 | 7.24 ± 1.03 mm | 4.11 ± 1.49 mm | NA | NA | NA | NA | NA | 87% > 35 N/cm | NA | NA | No conflict of interest reported |
|  |  |  |  |  |  | _ | Autogenous bone blocks | Augmentation with autogenous bone graft blocks. | 15 | 0 | 15 | 6 |  |  | 3.03 ± 0.66 mm | 6 | 7.46 ± 0.90 mm | 4.43 ± 0.78 mm | NA | NA | NA | NA | NA | 93% > 35 N/cm | NA |  |  |
|  | **Sanz et al. (2025)** | University; Multicenter | Spain, Norway, France, Denmark | Vertical and lateral ridge augmentation | RCT | MSCs | β-TCP/HA | Augmentation with MSCs incorporated into β-TCP/HA | 36 | 10 | 26 | 5.5 | •CBCT  •PROMS | Ridge bone volume | 3732 (SD = 1932) mm3 | 5 | 4799.13  (SD = 2448.4) mm3 | −1066.91 (−1360.00; −773.77) | NA | NA | NA | NA | Mesio-distal:  Site #1: 70.87 ± 7.68 Site #2: 69.74 ± 7.40  Bucco-lingual:  Site #1: 67.91 ± 9.83  Siite #2 69.43 ± 8.59 | NA | • Very satisfied  Test: 14 (53.8%)  Control: 5 (41.7%)  •Satisfied  Test: 8 (30.8%)  Control: 1 (50.0%)  •Neither satisfied nor dissatisfied  Test: 4 (15.4%)  Control: 1 (8.3%) | • During bone marrow aspiration: 1 patient minor hematoma (2-day sick leave). 1 patient pain and nausea • Surgery-related adverse events: 2 patients in the test group. 1 patient in the control group. All were minor.  • Late postoperative events (>2 weeks): 4 patients in the test group. 2 patients in the control group. Mostly sensory disturbances. | No conflict of interest reported |
|  |  |  |  |  |  | _ | Autogenous bone blocks | Augmentation with autogenous bone graft blocks. | 12 | 0 | 12 |  |  |  | 2739; (SD = 1165)mm3 | 5 | 3325.90(SD = 1559.6mm3 | −586.9 (−911.00; 262.69) | NA | NA | NA | NA | Mesio-distal:  Site #1: 70.09 ± 10.62  Site #2: 68.27 ± 14.49  Bucco-lingual:  Site #1: 70.36 ± 10.01  Site #2: 71.27 ± 10.87 | NA |  |  |  |
|  | **Thoma et al. (2018)** | University; Multicenter | Switzerland; Austria | Lateral ridge augmentation | RCT | rhBMP-2 | DBBM block and DBBM particles | Augmentation with DBBM block hydrated with rhBMP-2 and blocks fixed with one or two bone screws and xenograft particles added around the blocks. Collagen membranes were fixated with tacks | 12 | 0 | 11 | 4 | •Clinical measurements  •Histology •Questionnaire | RW | 3.18 ± 1.25 mm | 4 | 6.91 ± 1.64 mm | NA | NA | 29.8 | 31.5 | 9 | NA | NA | •Intra-oral sensitivity at suture removal (%): 58.00 ± 49.00 •Intra-oral sensitivity at 4 months (%): 91.00 ± 29.00 •Extra-oral sensitivity at suture removal (%): 58.00 ± 49.00 •Extra-oral sensitivity at 4 months (%): 92.00 ± 28.00 •Pain killer days (days): 2.73 ± 1.90 •VAS pain during surgery recipient site: 11.33 ± 13.55 •VAS pain during surgery donor site: 0 •VAS pain after surgery recipient site: 24.25 ± 20.08 •VAS pain after surgery donor site: 0 •VAS swelling after surgery recipient site: 42.00 ± 32.56 •VAS swelling after surgery donor site: 0 •VAS willingness to repeat the treatment: 93.54 ± 12.65 | NA | No conflict of interest reported |
|  |  |  |  |  |  | _ | Autogenous bone blocks and DBBM particles | Augmentation with autogenous bone harvest from symphysis or retromolar area. Collagen membranes were fixated with tacks. | 12 | 0 | 11 | 4 |  |  | 2.18 ± 0.87 mm | 4 | 6.73 ± 1.62 mm | NA | NA | 75.8 | 7.8 | 0 | NA | NA | •Intra-oral sensitivity at suture removal (%): 58.00 ± 49.00 •Intra-oral sensitivity at 4 months (%): 50.00 ± 50.00 •Extra-oral sensitivity at suture removal (%): 67.00 ± 47.00 •Extra-oral sensitivity at 4 months (%): 50.00 ± 50.00 •Pain killer days (days): 4.36 ± 2.73 •VAS pain during surgery recipient site: 31.00 ± 21.45 •VAS pain during surgery donor site: 29.25 ± 15.59 •VAS pain after surgery recipient site: 42.25 ± 27.81 •VAS pain after surgery donor site: 40.58 ± 29.02 •VAS swelling after surgery recipient site: 41.92 ± 35.90 •VAS swelling after surgery donor site: 38.00 ± 27.45 •VAS willingness to repeat the treatment: 82.33 ± 26.88 | NA |  |
|  | **Thor et al. (2005)** | University; Single center | Sweden | Vertical and lateral ridge augmentation | RCT | PRP | Autogenous bone particles | Augmentation with particulated autogenous bone mixed with PRP. | 19 | 0 | 19 | 12 | •RX •RFA | MBL | -1.3 ± 1.9 mm | 12 | - 1.8 ± 1.1 mm | NA | NA | NA | NA | NA | Higher in the test group (statistically significant at abutment connection) | NA | NA | • Two patients in all sample: infections | No conflict of interest reported |
|  |  |  |  |  |  | _ | Autogenous bone blocks | Augmentation with autogenous bone blocks. | 19 | 0 | 19 | 12 |  |  | -1.5 ± 1.7 mm | 12 | - 2.0 ± 0.9mm | NA | NA | NA | NA | NA | NA | NA | NA |  |  |
|  | **Torres et al. (2010)** | University and private practice; Multicenter | Spain | Vertical and lateral ridge augmentation | RCT | PRP | DBBM particles | Vertical and lateral bone augmentation with DBBM covered with PRP in addition to the Ti-mesh. | 15 | 0 | 15 | 6 | •Histology •CBCT | RH; RW | NA | 6 | NA | •RH: 3.5 ± 0.7 mm  •RW: 4.1 ± 0.6 mm | NA | NA | NA | NA | NA | NA | NA | • One patient in all sample: infection | No conflicts of interest reported.  Study funded by the Spanish Ministry of Science and Technology. |
|  |  |  |  |  |  | _ | DBBM particles | Vertical and lateral bone augmentation with DBBM covered by a Ti-mesh. | 15 | 0 | 15 | 6 |  |  | NA | 6 | NA | •RH: 3.1 ± 0.8 mm.   •RW: 3.7 ± 0.6 mm | NA | NA | NA | NA | NA | NA | NA |  |  |
|  | **Xie et al. (2024)** | University; Single center | China | Lateral ridge augmentation | RCT | GCF | DBBM particles | Dental implants were placed with simultaneous bone augmentation with sticky bone combined with CGF. | 14 | 0 | 14 | 6 | •Clinical measurements  •CBCT | Bone volume | NA | 6 | 72.09 ± 12.18% | NA | 1330.27± 311.50 HU | NA | NA | NA | NA | NA | NA | No | No conflict of interest reported This study was supported by the Xuzhou Science and Technology Bureau projects Grant Nos (KC23222). |
|  |  |  |  |  |  | _ | DBBM particles | Dental implants were placed with simultaneous bone augmentation with DBBM particles mixed with saline. | 14 | 0 | 14 | 6 |  |  | NA | 6 | 57.47 ± 9.62% | NA | 1240.47± 189.93 HU | NA | NA | NA | NA | NA | NA | No |  |
| **Alveolar ridge preservation** | **Abaza et al. (2024)** | University; Single center | Egypt | Ridge preservation | RCT | I-PRF | DBBM particles | Tooth extraction was performed and I-PRF with DBBM were placed into the extraction socket. | 12 | 0 | 12 | 12 | •Clinical measurements (caliper) •CBCT | RW | 9.23 ± 2.39 mm | 4 | 8.60 ± 1.27 mm | -0.63 ± 0.9 mm | NA | 28.74  ±  5.15 | NA | 6.76 ± 2.59 | NA | NA | NA | NA | NA |
|  |  |  |  |  |  | Hyaluronic acid | DBBM particles | Tooth extraction was performed and HA with DBBM were placed into the extraction socket. | 12 | 0 | 12 | 12 |  |  | 9.96 ± 2.45 mm | 4 | 9.78 ± 0.87 mm | -0.18 ± 0.28 mm | NA | 56.66  ± 7.35 | NA | 2.63 ± 1.27 | NA | NA | NA | NA |  |
|  |  |  |  |  |  | _ | DBBM particles | Tooth extraction was performed and I DBBM were placed into the extraction socket. | 12 | 0 | 12 | 12 |  |  | 9.26 ± 0.99 mm | 4 | 7.99 ± 0.89 mm | -1.27 ± 0.9 mm | NA | 24.05 ± 3.64 | NA | 2.71 ± 1.24 | NA | NA | NA | NA |  |
|  | **Areewong et al. (2019)** | University; Single center | Thailand | Ridge Preservation | RCT | PRF | FDBA | Tooth extraction was performed and FDBA covered with PRF were placed into the extraction socket. | 18 | 0 | 18 | 2 | • Histology | NA | NA | NA | NA | NA | NA | 31.33 | NA | NA | NA | NA | NA | NA | No conflict of interest reported |
|  |  |  |  |  |  | _ | FDBA | Tooth extraction was performed and FDBA covered with ACS were placed into the extraction socket. | 18 | 0 | 18 | 2 |  |  | NA | NA | NA | NA | NA | 26.33 | NA | NA | NA | NA | NA | NA |  |
|  | **Azangookhiavi et al. (2024)** | University; Single center | Iran | Ridge Preservation | RCT | PRF | FDBA | Tooth extraction was performed and FDBA covered with PRF were placed into the extraction socket; after 4 months implant was placed. | 20 | 0 | 20 | 6 | •Clinical measuraments •RX | NA | NA | NA | NA | NA | NA | NA | NA | NA | NA | NA | NA | NA | No conflict of interest reported |
|  |  |  |  |  |  | _ | FDBA | Tooth extraction was performed and FDBA covered with a collagen sponge were placed into the extraction socket, after 4 months implant was placed. | 20 | 0 | 20 | 6 |  |  | NA | NA | NA | NA | NA | NA | NA | NA | NA | NA | NA | NA |  |
|  | **Clark et al. (2018)** | University; Single center | USA | Ridge Preservation | RCT | A-PRF | None | Tooth extraction was performed and A-PRF material were placed into the extraction socket. | 12 | 2 | 10 | 3.5 | •Histology •CBCT • Clinical measurements (caliper) •Questionnaire | RH; RW | NA | 3 | NA | •RH: - 1.8mm ± 2.1 mm •RW: - 2.8 mm ± 1.2 mm | NA | 46 ± 18 | NA | NA | NA |  | •VAS pain after surgery recipient site (score 1-100): 24.25 ± 20.08 | NA | Authors received travel reimbursement from granting corporation |
|  |  |  |  |  |  | A-PRF | FDBA | Tooth extraction was performed and A-PRF with FDBA material were placed into the extraction socket. | 10 | 0 | 10 | 3.5 |  |  | NA | 3 | NA | •RH: - 1.0mm ± 2.3 mm •RW: - 1.9 mm ± 1.1 mm | NA | NA | NA | 3 ± 3 | NA | NA | •VAS pain after surgery donor site (score 1-100): 0 | NA |  |
|  |  |  |  |  |  | _ | FDBA | Tooth extraction was performed and FDBA material were placed into the extraction socket. | 12 | 2 | 10 | 3.5 |  |  | NA | 3 | NA | •RH: - 2.2 mm ± 1.8 mm •RW: - 2.5 mm ± 1.1 mm | NA | 29 ± 14 | NA | 11 ± 9 | NA | NA | •VAS swelling after surgery recipient site (score 1-100): 42.00 ± 32.56 | NA |  |
|  |  |  |  |  |  | _ | None | Tooth extraction was performed and sockets were left to heal naturally without any grafting material. | 11 | 1 | 10 | 3.5 |  |  | NA | 3 | NA | •RH: - 3.8mm ± 2.0 mm •RW: - 2.9 mm ± 1.7 mm | NA | NA | NA | NA | NA | NA | •VAS swelling after surgery donor site (score 1-100): 0 | NA |  |
|  | **Coomes et al. (2014)** | University; Single center | USA | Ridge Preservation | RCT | rhBMP-2 | ACS | Tooth extraction was performed and rhBMP-2/ACS carrier to a collagen sponge was placed in the extraction socket | 20 | 2 | 18 | 5 | •CBCT •Clinical measuraments •Questionnaire | RH; RW | NA | 5 | **•**RH Radiographic: 3.42 ± 1.46 mm  •RH Clinical: 6.81 ± 1.38 mm  •RW Clinical:6.0 ± 1.58 mm  •RW Radiographic: -2.07 ± 1.17mm | NA | NA | NA | NA | NA | NA | NA | •VAS willingness to repeat the treatment (score 1-100): 93.54 ± 12.65 | •Mild erythema and localized post- operative swelling 2 to 3 days after extraction (12%) | Disclosed. Dr. Cochran received funding for research, consulting, and lecturing from Medtronic and Institute Straumann. Other authors reported no conflicts. |
|  |  |  |  |  |  | _ | ACS | Tooth extraction was performed and collagen sponge was placed in the extraction socket | 19 | 3 | 16 | 5 |  |  | NA | 5 | •RH Radiographic: 5.16 ± 2.39 mm  •RH Clinical: 10.00 ± 3.51 mm  •RW Clinical: -3.40 ± 1.73 mm  •RW Radiographic: 4.48 ± 2.12 mm | NA | NA | NA | NA | NA | NA | NA | NA | •Mild erythema and localized post- operative swelling 2 to 3 days after extraction (0%) |  |
|  | **Fiorellini et al. (2005)** | University; Multicenter | USA | Ridge Preservation | RCT | 0.75 mg/mL rhBMP-2 | ACS | Tooth extraction was performed and a collagen sponge with 0.75 mg/mL rhBMP-2 was placed in the extraction socket | 20 | 0 | 22 | 4 | •CT •Histology | RH; RW | NA | NA | NA | •RH: −0.62 ± 1.39 mm •RW:1.76 ± 1.67 mm | NA | NA | NA | NA | NA | NA | NA | Most frequent adverse events in all sample:  •Oral edema (75%)  •Mouth pain (68%) •Oral erythema (46%) | No conflict of interest reported |
|  |  |  |  |  |  | 1.50 mg/mL rhBMP-2 | ACS | Tooth extraction was performed and a collagen sponge with 1.50 mg/mL rhBMP-2 was placed in the extraction socket | 20 | 0 | 21 | 4 |  |  | NA | NA | NA | •RH: 0.02 ± 1.20 mm •RW: 3.27 ± 2.53 mm | NA | NA | NA | NA | NA | NA | NA |  |  |
|  |  |  |  |  |  | _ | ACS | Tooth extraction was performed and a collagen sponge was placed in the extraction socket, | 20 | 0 | 17 | 4 |  | RH; RW | NA | NA | NA | •RH: −1.00 ± 1.40 mm •RW 0.82 ± 1.40 mm | NA | NA | NA | NA | NA | NA | NA |  |  |
|  |  |  |  |  |  | _ | None | The extraction socket underwent natural healing | 20 | 0 | 20 | 4 |  |  | NA | NA | NA | •RH: −1.17 ± 1.23 mm •RW: 0.57 ± 2.56 mm | NA | NA | NA | NA | NA | NA | NA |  |  |
|  | **Han et al. (2021)** | University; Multicenter | Korea | Ridge Preservation | RCT | rhBMP-2 | β-TCP/HA | Tooth extraction was performed and β-TCP/HA graft material coated with rhBMP-2 was placed in the extraction socket. | 36 | 0 | 36 | 3 | •CT | RH; RW | NA | NA | NA | •RH: −0.059 ± 0.960 mm •RW: 1.279±1.387 mm | NA | NA | NA | NA | NA | NA | NA | NA | No conflict of interest reported. The study was supported by the Korea Healthcare Technology R&D Project. |
|  |  |  |  |  |  | _ | β-TCP/HA | Tooth extraction was performed and β-TCP/HA graft material was placed in the extraction socket. | 36 | 0 | 36 | 3 |  |  | NA | NA | NA | •RH: −0.059 ± 0.960 mm •RW: 0.006 ±1.149 mm | NA | NA | NA | NA | NA | NA | NA | NA |  |
|  | **Kaigler al. (2013)** | University; Single center | USA | Lateral ridge augmentation | RCT | Autologous bone marrow-derived CD90- and CD14-positive cells. | ACS | Tooth extraction was performed and autologous bone marrow-derived CD90- and CD14-positive cells into a ACS were placed into the extraction socket. Patients were divided into two groups according to the follow-up: Group I: 1.5 month Group II: 3 months | 12 | 0 | 12 | •Group I: 1.5 month •Group II: 3 months | •Linear bone height (%) •Micro CT •Histology •Histomorphometric analysis | NA | NA | NA | Group I: 78.9%  Group II: 80.1% | NA | • Group I: 195.0 (mg/cc) • Group II:186.8 (mg/cc) | NA | NA | NA | NA | NA | NA | None | No conflict of interest reported. This study was funded by a Career Award for Medical Scientists from the Burroughs Wellcome Fund (D.K.), the National Institutes of Health NIH/NCRR UL1RR024986 (W.V.G.), and National Institutes of Health NIH/ NIDCR DE13397 (W.V.G.). |
|  |  |  |  |  |  | _ | ACS + collagen membrane | Tooth extraction was performed and an ACS was placed into the extraction socket. Patients were divided into two groups according to the follow-up: Group I: 1.5 month Group II: 3 months | 12 | 0 | 12 | •Group I: 1.5 month •Group II: 3 months |  |  | NA | NA | Group I: 55.3%  Group II: 74.6% | NA | • Group I: 85.5 (mg/cc) • Group II: 146.6 (mg/cc) | NA | NA | NA | NA | NA | NA | None |  |
|  | **Kim et al. (2014)** | University; Multicenter | Korea | Ridge Preservation | RCT | rhBMP-2 | Demineralized bone matrix gel | Tooth extraction was performed and rhBMP-2/demineralized bone matrix gel covered with a collagen matrix was placed in the extraction socket. | 35 | 6 | 29 | 3 | •CT | RH; RW | •RH: 20.98 ± 7.74 mm •RW: 8.06 ± 2.22 mm | 3 | •RH: 19.81 ± 7.73 mm •RW: 7.63 ± 2.22 mm | •RH: -1.17 ± 0.82 mm  •RW: −0.43 ± 0.71 mm | NA | NA | NA | NA | NA | NA | NA | None | No conflict of interest reported. |
|  |  |  |  |  |  | _ | Demineralized bone matrix gel | Tooth extraction was performed and demineralized bone matrix gel covered with a collagen matrix was placed in the extraction socket. | 34 | 4 | 30 | 3 |  |  | •RH: 21.83 ± 6.89 mm •RW: 8.57 ± 2.44 mm | 3 | •RH: 20.32 ± 6.92 mm •RW: 7.98 ± 2.34 mm | •RH: -1.50 ± 1.07 mm •RW: −0.58 ± 0.68mm | NA | NA | NA | NA | NA | NA | NA | None |  |
|  | **Kumar et al. 2018** | University; Single center | India | Ridge preservation | RCT | PRF | Plaster of Paris | Tooth extraction was performed and POP covered with PRF plugs was placed in the extraction socket. | NA | NA | NA | 6 | •RX •Questionnaire | RH; RW | •RH: 8.3 ± 1.18 mm •RW 9.6 ± 1.47 mm | 6 | •RH: 5.6 ± 1.13 mm •RW: 6.78 ± 1.26 mm | •RH: 2.8 ± 0.46 mm •RW: 2.9 ± 0.8 mm | NA | NA | NA | NA | NA | NA | NA | 3 patients (10%) who had swelling 24 h post- operatively | No conflict of interest reported |
|  |  |  |  |  |  | PRF | None | Tooth extraction was performed and PRF was placed in the extraction socket. | NA | NA | NA | 6 |  |  | •RH: 8.5 ± 1.22 mm •RW 9.7 ± 1.73 mm | 6 | •RH: 5.5 ± 1.04 mm  •RW: 6.7 ± 1.33 mm | •RH: 3 ± 0.8 mm •RW: 3 ± 0.64 mm | NA | NA | NA | NA | NA | NA | NA | No swelling post-extraction. |  |
|  |  |  |  |  |  | _ | None | Extraction socket underwent spontaneous healing. | NA | NA | NA | 6 |  |  | •RH: 8.43 ± 1.13 mm •RW: 8.16 ± 1.87 mm | 6 | •RH: 5.0 ± 0.86 mm •RW: 5.2 ± 1.59 mm | •RH: 3.3 ± 0.61 mm •RW: 3 ± 0.83 mm | NA | NA | NA | NA | NA | NA | NA | 1 patient (6%) had swelling 24 h post- operatively. |  |
|  | **Lin et al. (2021)** | University; Single center | China | Ridge preservation | RCT | CGFs | DBBM | Tooth extraction was performed and CGFs combined with DBBM, covered with CGFs membrane, was placed in the extraction socket. | 12 | 0 | 12 | 8 | •CBCT •Histology | NA | NA | NA | NA | NA | NA | 41.99 ± 12.99 | NA | 16.19 ± 6.63 | NA | NA | NA | None | No conflict of interest reported. This work was supported by the Science and Technology Project of the State Grid Corporation of China (Grant No. 52720016004N). |
|  |  |  |  |  |  | _ | DBBM | Tooth extraction was performed and DBBM alone, covered with a collagen membrane, was placed in the extraction socket. | 12 | 0 | 12 | 8 |  |  | NA | NA | NA | NA | NA | 30.68 ± 10.95 | NA | 28.35 ± 11.70 | NA | NA | NA | None |  |
|  |  |  |  |  |  | _ | None | Extraction socket underwent spontaneous healing. | 12 | 0 | 12 | 8 |  |  | NA | NA | NA | NA | NA | NA | NA | NA | NA | NA | NA | 0 |  |
|  | **Mercado et al. (2021)** | University; Multicenter | Australia | Ridge Preservation | RCT | EMD | DBBMC | Tooth extraction was performed and EMD with DBBMC was placed in the extraction socket. | 21 | 0 | 21 | 4 | •CBCT •Histology •Histomorphometric analysis | RH; RW | • RH: 17.4 ± 1.9 mm • RW: 7.2 ± 1.0 mm | 4 | •RH: 16.7 ± 2.2 mm  •RW: 6.6 ± 1.1 mm | •RH: −0.62 ± 0.9 mm  •RW: −0.40 ± 0.5 mm | NA | 45.1 ± 8.8 | 34.6 ± 13.8 | 20.3 ± 7.2 | NA | NA | NA | NA | No conflict of interest reported. FM was supported by a scholarship from the National Health and Medical Research Council Australia |
|  |  |  |  |  |  | _ | DBBMC | Tooth extraction was performed and DBBMC was placed in the extraction socket. | 21 | 0 | 21 | 4 |  |  | • RH: 17.6 ± 3.3 mm • RW: 7.7 ± 0.96 mm | 4 | •RH: 16.5 ± 3.7 mm •RW: 6.8 ± 1.3 mm | •RH: −1 ± 1.2 mm  •RW: −0.37 ± 1.1 mm | NA | 16.5 ± 6.9 | 46.5 ± 10.4 | 36.8 ± 8.8 | NA | NA | NA | NA |  |
|  | **Stumbras et al. (2020)** | University; Multicenter | Lithuania, Spain | Ridge Preservation | RCT | PRGF | DBBM | Tooth extraction was performed and the extraction socket was filled with PRGF + DBBM covered with a resorbable native collagen membrane. | 11 | 1 | 10 | 3 | •Histomorphometric analysis | NA | NA | 3 | NA | NA | NA | 20.3 ± 21.9 | 34.7 ± 10.5 | 45 ± 19 | NA | NA | NA | NA | No conflict of interest reported |
|  |  |  |  |  |  | PRGF | FDBA | Tooth extraction was performed and the extraction socket was filled with PRGF + FDBA covered with a resorbable native collagen membrane. | 11 | 1 | 10 | 3 |  |  | NA | 3 | NA | NA | NA | 7.2 ± 8.6 | 54.3 ± 18.5 | 38.6 ± 26.4 | NA | NA | NA | NA |  |
|  |  |  |  |  |  | PRGF | None | Tooth extraction was performed and the extraction socket was filled with PRGF. | 11 | 1 | 10 | 3 |  |  | NA | 3 | NA | NA | NA | 75.5 ± 16.3 | 24.5 ± 16.3 | NA | NA | NA | NA | NA |  |
|  |  |  |  |  |  | _ | None | Tooth extraction was performed and the extraction socket underwent natural healing. | 10 | 0 | 15 | 3 |  |  | NA | 3 | NA | NA | NA | 46.5 ± 15.2 | 53.5 ± 15.2 | NA | NA | NA | NA | NA |  |
|  | **Stumbras et al. (2021)** | University; Multicenter | Lithuania, Spain | Ridge Preservation | RCT | PRGF | DBBM | Tooth extraction was performed and the extraction socket was filled with PRGF + DBBM covered with a resorbable native collagen membrane. | 11 | 1 | 10 | 3 | •CBCT | RH; RW | •RH: 10.81 ± 2.18 mm •RW: 7.89 ± 1.11 mm | 3 | •RH: 10.55 ± 2.38 mm •RW: 7.22 ± 0.86 mm | •RH: −0.26 ± 0.91 mm •RW: −0.68 ± 0.67 mm | NA | NA | NA | NA | NA | NA | NA | NA | No conflict of interest reported |
|  |  |  |  |  |  | PRGF | FDBA | Tooth extraction was performed and the extraction socket was filled with PRGF + FDBA covered with a resorbable native collagen membrane. | 11 | 1 | 10 | 3 |  |  | •RH: 11.54 ± 2.87 mm •RW: 8.23 ± 2.19 mm | 3 | •RH: 10.86 ± 2.63 mm •RW: 6.73 ± 1.96 mm | •RH: −0.68 ± 0.85 mm •RW: −1.49 ± 0.87 mm | NA | NA | NA | NA | NA | NA | NA | NA |  |
|  |  |  |  |  |  | PRGF | None | Tooth extraction was performed and the extraction socket was filled with PRGF. | 11 | 1 | 10 | 3 |  |  | •RH: 9.56 ± 3.26 mm •RW: 7.75 ± 1.37 mm | 3 | •RH: 9.03 ± 2.92 mm •RW: 7.22 ± 2.23 mm | •RH: −0.54 ± 0.86 •RW:−1.25 ± 1.10 mm | NA | NA | NA | NA | NA | NA | NA | NA |  |
|  |  |  |  |  |  | _ | None | Tooth extraction was performed and the extraction socket underwent natural healing. | 10 | 0 | 15 | 3 |  |  | •RH: 7.99 ± 2.63 mm •RW: 7.59 ± 1.6 mm | 3 | •RH: 7.13 ± 2.46 mm •RW: 5.99 ± 0.73 mm | •RH: −0.86 ± 0.43 mm •RW: −1.61 ± 1.7 mm | NA | NA | NA | NA | NA | NA | NA | NA |  |
| **Sinus floor elevation** | **Barros-Mourão CF et al. 2019** | University; Single center | Brazil | Lateral | RCT | BDGF | Nanostructured carbonated hydroxyapatite (cHA) microspheres | The sinus cavity was grafted with cHA with BDGF. | 10 | 0 | 10 | 6 | •CBCT | RH | NA | NA | NA | NA | NA | NA | NA | NA | NA | NA | NA | NA | No conflict of interest reported. |
|  |  |  |  |  |  | _ | Nanostructured carbonated hydroxyapatite (cHA) microspheres | The sinus cavity was grafted with cHA with 0.9% saline solution. | 10 | 0 | 10 | 6 |  |  | NA | NA | NA | NA | NA | NA | NA | NA | NA | NA | NA | NA |  |
|  | **Bettega et al. (2009)** | University; Single center | France | Lateral | CT | APCs | Autogenous bone particles | The sinus cavity was grafted with with 2 mL of cancellous bone and APCs. | 18 | 6 | 12 | 6 | •Clinical measurements •RX •CT •Histology | RH | Median: 3.50 (2; 5.25) mm | 6 | Median: 11.5 (9.5; 16) mm | NA | 88.9 | Median: 37.1 (31.15; 48.1) mm | NA | NA | NA | NA | NA | NA | No conflict of interest reported.  The study was funded by the Délégation Régionale à la Recherche Clinique de Grenoble. |
|  |  |  |  |  |  | _ | Autogenous bone blocks | The sinus cavity was grafted with iliac crest bone alone. | 19 | 6 | 12 | 6 |  |  | Median: 3.0 (1.75; 6.25) mm | 6 | Median: 10 (8.5; 16.25) mm | NA | 72.2 | Median: 42.5 (28.25; 51.15) mm | NA | NA | NA | NA | NA | NA |  |
|  | **Boyne et al. (2005)** | University and private practice; Multicenter | USA | Lateral | RCT | 0.75 mg/mL rhBMP-2 | ACS | The sinus cavity was grafted with 0.75 mg/mL rhBMP-2/ACS. | 18 | NA | 18 | 4 | •CT •Histology | RH; RW | <6 mm | 4 | NA | •RH: 9.47 ± 5.72 mm  •RW: 2.02 ± 2.70 mm | NA | NA | NA | 0 | NA | NA | NA | •Dehiscence: 2 (11%) •Edema: 0 •Face edema: 7 (39%) •Headache: 2 (11%) •Pain: 1 (6%) •Mouth pain: 14 (78%) •Oral edema:10 (56%) •Oral erythema: 3 (17%) •Ecchymosis: 6 (33%) •Arthralgia: 0 •Bone disorder: 4 (22%) •Sinusitis: 4 (22%) •Rash: 2 (11%) | No conflict of interest reported. Study funded by Wyeth/Genetics Institute. One author was affiliated with the sponsor, |
|  |  |  |  |  |  | 1.50 mg/mL rhBMP-2 | ACS | The sinus cavity was grafted with 1.50 mg/mL rhBMP-2/ACS. | 17 | NA | 17 | 4 |  |  | NA | 4 | NA | •RH: 16.00 ± 4.70 mm •RW: 1.98 ± 2.41 mm | NA | NA | NA | 0 | NA | NA | NA | •Dehiscence:1 (6%) •Edema: 0 •Face edema: 14 (82%) •Headache: 3 (18%) •Pain: 3 (18%) •Mouth pain: 15 (88%) •Oral edema: 8 (47%) •Oral erythema: 4 (24%) •Ecchymosis: 3 (18%) •Arthralgia 2 (11%) •Bone disorder: 2 (11%) •Sinusitis: 1 (6%) •Rash: 1 (6%) |  |
|  |  |  |  |  |  | _ | Standard autograft (with or without allograft ) | The sinus cavity was grafted with standard autograft. | 13 | NA | 13 | 4 |  |  | NA | 4 | NA | •RH: 11.29 ± 4.12 mm •RW: 4.66 ± 2.75 mm | NA | NA | NA | NA | NA | NA | NA | •Dehiscence: 2 (15%) •Edema: 6 (46%) •Face edema: 5 (38%) •Headache: 0 •Pain: 5 (38%) •Mouth pain: 8 (62%) •Oral edema: 8 (62%) •Oral erythema: 6 (46%) •Ecchymosis: 3 (23%) •Arthralgia: 3 (23%) •Bone disorder: 1(8%) •Sinusitis: 4 (31%) •Rash: 6 (46%) |  |
|  | **Fatale et al. (2022)** | University; Single center | Italy | Lateral and crestal | CT | MSC | β-TCP/HA | Crestal-approach sinus lift with immediate implant placement or a lateral-approach sinus lift was performed, graft. The graft used was composed of beta-tricalcium phosphate (80%), hydroxyapatite (20%), tetracycline and collagen particles. | 12 | 0 | 12 | 3 | •Histology •Histomorphometric analysis | NA | NA | NA | NA | NA | NA | Type 1 tissue: 44.45 Type 2 tissue: 7.04 | NA | NA | NA | NA | Some patients complained of mild and painless swelling on the first day after the surgery, no patient reported any major intraoperative or postoperative complications | NA | NA |
|  |  |  |  |  |  | _ | β-TCP/HA | Crestal-approach sinus lift with immediate implant placement or a lateral-approach sinus lift was performed, graft. The graft used was composed of beta-tricalcium phosphate (80%), hydroxyapatite (20%), tetracycline, collagen particles, and MSCs, which were added to the synthetic graft material prior to graft placement. | 12 | 0 | 12 | 3 |  | NA | NA | NA | NA | NA | NA | Type 1 tissue: 27.24 Type 2 tissue: 10.86 | NA | NA | NA | NA |  | NA |  |
|  | **Froum et al. (2013)** | University; Single center | USA | Lateral | RCT | rhBMP-2 | ACS; mineralized particulate cancellous allograft | The sinus cavity was grafted with mineralized cancellous bone allograft and 8.4 mg of rhBMP-2/ACS (5.6 mL of rhBMP-2/ACS). | NA | NA | NA | 6 to 9 | •Histology | NA | NA | 6 to 9 | NA | NA | NA | 25.3 ± 15.3 | NA | 22.6 ± 7.0 | NA | NA | NA | NA | No conflict of interest reported. Study supported by Medtronic Sofamor Danek USA. |
|  |  |  |  |  |  | rhBMP-2 | ACS; mineralized particulate cancellous allograft | The sinus cavity was grafted with mineralized cancellous bone allograft and 4.2 mg of rhBMP-2/ACS (2.8 mL of rhBMP-2/ACS). | NA | NA | NA | 6 to 9 |  |  | NA | 6 to 9 | NA | NA | NA | 17.5 ± 10.9 | NA | 10.5 ± 12.8 | NA | NA | NA | NA |  |
|  |  |  |  |  |  | _ | Mineralized particulate cancellous allograft | The sinus cavity was grafted with mineralized cancellous bone allograft only. | NA | NA | NA | 6 to 9 |  |  | NA | 6 to 9 | NA | NA | NA | 21.5 ± 11.6 | NA | 23.2 ± 12.9 | NA | NA | NA | NA |  |
|  | **Froum et al. (2013)** | University; Single center | USA | Lateral | RCT | rhPDGF-BB | DBBM particles | The sinus cavity was grafted with DBBM and rhPDGF-BB. The sample was divided in two groups according to the follow-up:  Group A: 4.25 ± 0.34 months. Group B: 8.13 ± 0.53 months. | 24 | 0 | 12 | From 4.25 ± 0.34 to 8.13 ± 0.53 m | •CT •Histology | NA | NA | From 4.25 ± 0.34 to 8.13 ± 0.53 m | NA | NA | NA | 20.3 ± 11.0 | 47.8 ± 10.7 | 30.2 ± 11.6 | NA | NA | NA | NA | No conflict of interest reported. Study sponsored by Luitpold Pharmaceuticals, but no explicit conflicts of interest were reported. |
|  |  |  |  |  |  | _ | DBBM particles | The sinus cavity was grafted with DBBM. The sample was divided in two groups according to the follow-up:  Group A: 4.25 ± 0.34 months. Group B: 8.13 ± 0.53 months. | 24 | 0 | 12 | From 4.25 ± 0.34 to 8.13 ± 0.53 m |  |  | NA | From 4.25 ± 0.34 to 8.13 ± 0.53 m | NA | NA | NA | 16.6 ± 10.0 | 46.3 ± 15.2 | 36.8 ± 10.2 | NA | NA | NA | NA |  |
|  | **Kaigler et al. (2015)** | University; Single center | USA | Lateral | RCT | Autologous bone marrow-derived cells enriched with CD90+ MSCs and CD14+ monocytes | β-TCP | The sinus cavity was grafted with β-TCP loaded with CD90+ MSCs cells and CD14+ monocytes. | 15 | 2 | 13 | 4 | •Clinical measurements •RX •Micro CT •Histology | RH | NA | 4 | NA | 12.2 ± 3.3 mm | 0.78 ± 0.02 mg/mm3 | NA | NA | NA | NA | NA | NA | NA | No conflicts of interest reported. |
|  |  |  |  |  |  | _ | β-TCP | The sinus cavity was grafted with β-TCP scaffold alone. | 15 | 2 | 13 | 4 |  |  | NA | 4 | NA | 12.8 ± 2.8 mm | 0.79 ± 0.05 mg/mm3 | NA | NA | NA | NA | NA | NA | NA |  |
|  | **Karagah et al. (2022)** | University; Single center | Iran | Lateral | RCT | L-PRF and PRF membrane | None | The sinus cavity was grafted with PRF and L-PRF membrane. | 10 | 0 | 10 | 6 | • Resonance frequency analysis | NA | NA | 6 | NA | NA | NA | NA | NA | NA | Baseline: 53.00 (2.384) 6 months: 69.85 (2.059) | NA | NA | NA | No conflict of interest reported |
|  |  |  |  |  |  | _ | FDBA | The sinus cavity was grafted with FDBA and collagen membrane. | 10 | 0 | 10 | 6 |  |  | NA | 6 | NA | NA | NA | NA | NA | NA | Baseline: 53.25 (2.268) 6 months: 62.65 (2.110) | NA | NA | NA |  |
|  | **Khairy et al. (2013)** | University; Single center | Egypt | Lateral | RCT | PRP | Autogenous bone graft | Maxillary sinus lifting with autogenous bone graft mixed with PRP, further divided into: Group IIA: Implant placement at 6 months. Group IIB: Implant placement at 4 months. | 10 | 0 | 10 | Group IIA: 6  Group IIB: 4 | •RX •Histomorphometric analysis •Histology | NA | NA | Group IIA: 6  Group IIB: 4 | NA | NA | •Group IIA: 136.7 ± 18.4 (pixels density)  •Group IIB: 155.8 ± 11.5 (pixels density) | NA | NA | NA | NA | NA | NA | NA | No conflict of interest reported |
|  |  |  |  |  |  | _ | Autogenous bone graft | Maxillary sinus lifting with autogenous bone graft, implant placement after 6 months. | 5 | 0 | 5 | 6 |  |  | NA | 6 | NA | NA | 139.8 ± 20.2 (pixels density) | NA | NA | NA | NA | NA | NA | NA |  |
|  | **Kim et al. (2015)** | University; Multicenter | South Korea | Lateral | RCT | rhBMP-2 | HA particles | The sinus cavity was grafted with rhBMP-2 combined with HA granules | 73 | 8 | 65 | 3 | •Histomorphometric analysis | NA | NA | 3 | NA | NA | NA | 16.10 ± 10.52 | 25.27 ± 10.86 | 58.64 ± 14.61 | NA | NA | NA | •8 patients prolonged swelling for >2 wk | No conflicts of interest reported. |
|  |  |  |  |  |  | _ | DBBM particles | The sinus cavity was grafted with DBBM | 74 | 12 | 62 | 3 |  |  | NA | 3 | NA | NA | NA | 8.25 ± 9.47 | 29.44 ± 15.31 | 62.31 ± 14.57 | NA | NA | NA | NA |  |
|  | **Lee et al. (2009)** | Private practice; Single center | USA | Lateral | RCT | PRP | Autogenous bone graft + DBBM particles | The sinus cavity was grafted with PRP sprayed autogenous bone combined with DBBM. | NA | 0 | NA | 6 | •Histomorphometric analysis •Histology | NA | NA | 6 | NA | NA | NA | 100 | NA | 6 | NA | NA | NA | NA | No conflict of interest reported |
|  |  |  |  |  |  | PRP | Autogenous bone graft + FDBA | The sinus cavity was grafted with PRP sprayed autogenous bone combined with FDBA | NA | 0 | NA | 6 |  |  | NA | 6 | NA | NA | NA | 77-98 | NA | 0 | NA | NA | NA | NA |  |
|  |  |  |  |  |  | PRP | Autogenous bone graft + FHA | The sinus cavity was grafted with PRP sprayed autogenous bone combined with FHA. | NA | 0 | NA | 6 |  |  | NA | 6 | NA | NA | NA | 100 | NA | 9 | NA | NA | NA | NA |  |
|  | **Lv et al. (2022)** | University; Single center | China | Crestal/Lateral | RCT | PRP | DBBM particles | Flapless osteotome-mediated sinus floor elevation using PRF. | 20 | 0 | 19 | 18 | •CBCT •Questionnaire | RH | 3.35 ± 0.79 mm | 18 | 7.67 ± 1.29 mm | NA | 668.88 ± 112.03 H.U. | NA | NA | NA | NA | NA | **•**Willing to undergo the surgery again: 18  •Do not reject, but with little anxious and fear: 2 •Do not reject, but with severe anxious and fear:0 •Unwilling to undergo the same surgery anymore: 0 VRS 0 -swelling at day: 0: 16 (80%)  1: 11 (55%) 2: 16 (80%) 3: 20 (100%) 7: 20 (100%) | NA | No conflict of interest reported. This work was supported by NationalNature Science Foundation of China(82170998, 82071152) and Key Scientificand Technological Projects of JilinProvince, China (No. 20170204041GX |
|  |  |  |  |  |  | _ | DBBM particles | Lateral sinus floor elevation using DBBM. | 20 | 0 | 20 | 18 |  |  | 2.92 ± 0.63 mm | 18 | 10.32 ± 1.26 mm | NA | 921.01 ± 130.61 H.U. | NA | NA | NA | NA | NA | **•**Willing to undergo the surgery again: 5 •Do not reject, but with little anxious and fear: 9 •Do not reject, but with severe anxious and fear: 4 •Unwilling to undergo the same surgery anymore: 2 VRS 0 -swelling at day: 0: 6 (30%) 1: 0 2: 0 3: 0 7: 12 (60%) | NA |  |
|  | **Merli et al. (2022)** | Private practice; Single center | Italy | Lateral | CT | CGFs | None | Sinus floor elevation using CGFs as the sole grafting material with simultaneous implant placement. | 10 | 0 | 20 | 12 | •RX •Questionnaire | RH (at implant) | 9.7 ± 1.9 mm | 12 | 9.0 **±** 2.1 mm | –0.7 ± 1.1 mm | NA | NA | NA | NA | NA | NA | VAS satisfaction: 9.7 ± 0.3 | NA | No conflicts of interest were reported. |
|  |  |  |  |  |  | _ | DBBM | Sinus floor elevation using DBBM as the grafting material with simultaneous implant placement. | 10 | 0 | 20 | 12 |  |  | 9.4 ± 1.1 mm | 12 | 8.9 ± 1.9 mm | –0.5 ± 1.0 mm | NA | NA | NA | NA | NA | NA | VAS satisfaction: 9.9 ± 0.4 | NA |  |
|  | **Nizam et al. (2017)** | University; Single center | Turkey | Lateral | RCT | L-PRF | DBBM | Sinus augmented with L-PRF + DBBM. | 13 | 0 | 13 | 6 | •RX •Histology •Histomorphometric analysis | RH | 2.45 ± 0.79 mm | 6 | 13.60 ± 1.09 mm | NA | NA | 21.38 ± 8.78 | 52.67 ± 12.53 | 25.95 ± 9.54 | NA | NA | NA | NA | No conflict of interest reported |
|  |  |  |  |  |  | _ | DBBM | Sinus augmented with DBBM. | 13 | 0 | 13 | 6 |  |  | 2.53 ± 0.61 mm | 6 | 13.53 ± 1.20 mm | NA | NA | 21.25 ± 5.59 | 45.96 ± 8.36 | 32.79 ± 5.89 | NA | NA | NA | NA |  |
|  | **Pichotano et al. (2019)** | University; Single center | Brazil | Lateral | RCT | L-PRF | DBBM particles | The sinus cavity was grafted with LPRF + DBBM; after 4 months dental implant was placed. | 12 | 0 | 12 | 4 | •CBCT  •Histology •RFA | Graft volume | 1.68 ± 0.42 cm3 | 4 | 1.10 ± 0.25 cm3 | 0.58 ± 0.26 cm3 | NA | 26.60 ± 11.13 | 44.58 ± 13.90 | 3.59 ± 4.22 | Baseline: 60.9 (9.35) Loading: 76.08 (5.86) | NA | NA | NA | No conflicts of interest were reported. Geistlich Pharma AG (Wolhusen, Switzerland), Grant/Award Number: Donation of Bio-Oss and BioGide; Neodent (Curitiba, PR, Brazil), Grant/Award Number: Donation of the implants; CAPES (Coordenação de Aperfeiçoamento de Pessoal de Nível Superior). |
|  |  |  |  |  |  | _ | DBBM particles | The sinus cavity was grafted with DBBM; after 8 months dental implant was placed. | 12 | 0 | 12 | 8 |  |  | 1.46 ± 0.53 cm3 | 8 | 0.91 ± 0.35 cm3 | 0.55 ± 0.34 cm3 | NA | 30.64 ± 12.46 | 30.02 ± 8.42 | 13.75 ± 9.99 | Baseline: 75.13 (5.69) Loading: 75.75 (6.14) | NA | NA | NA |  |
|  | **Rickert et al. (2011, 2014)** | University; Multicenter | Netherlands; Germany | Lateral | RCT | Bone marrow concentrate enriched in MSC | DBBM particles | The sinus cavity was grafted with DBBM seeded with bone marrow concentrate with isolated MSCs from the iliac crest; after 3-4 months dental implant was placed. | 12 | 0 | 12 | 3 (histologic analysis), 12(clinical records) | •Clinical measurements  •Histologic analysis •RX •Questionnaire | NA | 2.1 ± 0.3 | NA | NA | NA | NA | 13.9 | 52.5 | 31.3 | NA | NA | •Patient satisfaction with functioning of the maxillary denture after treatment was high in both groups. | NA | No conflicts of interest were reported. |
|  |  |  |  |  |  | _ | DBBM particles | The sinus cavity was grafted with DBBM mixed with autogenous bone; after 3-4 months dental implant was placed. | 12 | 0 | 12 | 3 (histologic analysis), 12(clinical records) |  |  | 2.2 ± 06 | NA | NA | NA | NA | 13.2 | 52.9 | 25.6 | NA | NA |  | NA |  |
|  | **Sauerbier et al. (2010)** | University; Multicenter | Germany | Lateral | RCT | BMAC | DBBM particles | The sinus cavity was grafted with DBBM and BMAC. | NA | NA | 25 | 3.5 | •CT •Histology •Histomorphometric analysis •Questionnaire | Radiographic bone volume | NA | NA | NA | 1.74 ± 0.69 mL | NA | 12.6 ± 1.7 | NA | 31.3 ± 2.7 | NA | NA | All patients recovered well from the surgical procedure. No major intraoperative complications occurred. No occurrence of pain, hematoma, or infection at any time after bone marrow aspiration and sinus floor augmentation in the postoperative time were recorded. | 0 | The laboratory work was financially supported by the Camlog Foundation, Basel, Switzerland. Technical support was given by Geistlich Biomaterials, Wolhusen, Switzerland and Harvest Technologies, Munich, Germany. |
|  |  |  |  |  |  | _ | DBBM particles + autogenous bone particles | The sinus cavity was grafted with a mixture of 70% BBM and 30% Autogenous bone particles | NA | NA | 11 | 3.5 |  |  | NA | NA | NA | 1.33 ± 0.62 mL | NA | 14.3 ± 1.8 | NA | 19.3 ± 2.5 | NA | NA | One patient suffered from an injury of the inferior alveolar nerve, which resulted from the harvesting of the retromolar bone. Two patients of this group had an infection of the bone harvesting site. All noticed complications healed within reasonable time and standard care. | 0 |  |
|  | **Taschieri et al. (2016)** | University; Single center | Italy | Lateral | CT | PRP | DBBM/β-TCP and HA | The sinus cavity was grafted with PRP with either a DBBM or β-TCP and Hydroxyapatite scaffold. | 20 | 0 | 10 | 12 | •Histology •Histomorphometric analysis | NA | NA | 12 | NA | NA | NA | NA | NA | NA | NA | NA | NA | NA | No conflict of interest reported |
|  |  |  |  |  |  | _ | DBBM | The sinus cavity was grafted with DBBM or β-TCP and Hydroxyapatite scaffold. | 20 | 0 | 10 | 12 |  |  | NA | 12 | NA | NA | NA | NA | NA | NA | NA | NA | NA | NA |  |
|  | **Testori et al. (2019)** | University ; Multicenter | Italy | Crestal | Prospective clinical study | L-PRF | None | Crestal sinus floor elevation with L-PRF was used as the sole grafting material. | 53 | 2 | 51 | 8 | •Clinical measurements | NA | NA | NA | NA | NA | NA | NA | NA | NA | NA | Ranging from 20 Ncm to 30 Ncm | NA | NA | No conflict of interest reported |
|  | **Triplett et al. (2009)** | University and private practice; Multicenter | USA | Lateral | RCT | rhBMP-2 | ACS | The sinus cavity was grafted with rhBMP-2/ACS. | 82 | 0 | 82 | 6 | •Clinical measurements •CT •Histology •Histomorphometric analysis | RH | NA | 6 | NA | 7.83 ± 3.52 mm | 200 (mg/cm³) | NA | NA | NA | NA | NA | NA | NA | No conflict of interest reported |
|  |  |  |  |  |  | _ | Autogenous bone particles | The sinus cavity was grafted with autogenous bone particles. | 78 | 0 | 78 | 6 |  |  | NA | 6 | NA | 9.46 ± 4.11 mm | 270 (mg/cm³) | NA | NA | NA | NA | NA | •Many patients complained of pain at the harvest site | •Sensory loss in 17% |  |
|  | **Torres et al. (2009)** | Private practice; Single center | Spain | Lateral | RCT | PRP | DBBM particles | The sinus cavity was grafted with DBBM and PRP. | 87 | NA | 5 | 6 | •Clinical measurements •RX •CT •Histology •Histomorphometric analysis | RH | NA | 6 | 10.4 ± 0.7 mm | NA | NA | 31 ± 5 | NA | 22.9 ± 2.5 | NA | NA | NA | NA | No conflict of interest reported |
|  |  |  |  |  |  | _ | DBBM particles | The sinus cavity was grafted with DBBM only. | 87 | NA | 5 | 6 |  |  | NA | 6 | 9.4 ± 0.7 mm | NA | NA | 21.3 ± 4.5 | NA | NA | NA | NA | NA | NA |  |
|  | **Tzur et al. (2021)** | Private practice; Single center | Israel | Lateral / bone void in jaw | Prospective clinical study | MSCs | HA particles | Sinus augmentation or jaw bone void enucleation was performed followed by BF transplantation into the prepared site. | 14 | 3 | 11 | 11 | •CBCT •Histology | RH | •Bone void: 2.9 mm  •Sinus: 3.6 mm | 6 | •Bone void: 15.7 mm  •Sinus: 15.9 mm | •Bone void: NA  •Sinus: 11.1 mm | NA | NA | NA | NA | NA | NA | NA | •After the liposuction aspiration, some of the participants experienced minor pain and discomfort as well as some redness and bruising.  •Minor pain andd edema that lasted 1-2 days after surgery | The study was sponsored and financially supported by Bonus BioGroup Ltd., the company that developed BonoFill. |
|  | **Vincent-Bugnas et al. (2020)** | University; Single center | France | Lateral | RCT | EMD | DBBM | The sinus cavity was grafted with EMD + DBBM. | 16 | 0 | 16 | 6 | •CBCT •Histology | RH | 2.6 mm | 6 | 12.5 ± 1.2 mm | NA | NA | 22.6 ± 5.2 | 43.1 ± 9.4 | 20.1 ± 10 | NA | NA | NA | NA | Institut Straumann provided materials and histology services for this study. Benjamin Pippenger and Michel Dard are employed by Institut Straumann. |
|  |  |  |  |  |  | _ | DBBM | The sinus cavity was grafted with DBBM. | 16 | 0 | 16 | 6 |  |  | 2.9 mm | 6 | 12.9 ± 0.7 mm | NA | NA | 15.5 ± 6.9 | 37.2 ± 16.5 | 20.0 ± 11.7 | NA | NA | NA | NA |  |
|  | **Wiltfang et al. (2003)** | University; Single center | Germany | Lateral | RCT | PRP | β-TCP | The sinus cavity was grafted with β-TCP with PRP. | 17 | 0 | 22 | 6 | •RX •Histology | NA | NA | 6 | NA | NA | NA | NA | NA | 13.8 | NA | NA | NA | NA | The study was supported by Curasan AG, but no explicit conflicts of interest were reported. |
|  |  |  |  |  |  | _ | β-TCP | The sinus cavity was grafted with β-TCP. | 18 | 0 | 23 | 6 |  |  | NA | 6 | NA | NA | NA | NA | NA | 15 | NA | NA | NA | NA |  |
| **Staged implant plancement** | **Elsheikh et al. (2023)** | University; Single center | Egypt | Implant placement in fresh sockets | RCT | PRF | None | Immediate implants were placement into fresh extraction sockets combined with PRF into the gap | 12 | 0 | 12 | 18 | •CBCT  •RFA | MBL | NA | 18 | 2.42 ± 0.46 mm | NA | NA | NA | NA | NA | Baseline: 64.33 ± 2.77 18 months: 73.50 ± 2.07 | NA | NA | NA | No conflict of interest reported Open access funding provided by The Science, Technology & Innovation Funding Authority (STDF) in cooperation with The Egyptian Knowledge Bank (EKB). The authors received no funding for this research |
|  |  |  |  |  |  | _ | DBBM particles | Immediate implants were placement into fresh extraction sockets combined with DBBM material into the gap | 12 | 0 | 12 | 18 |  |  | NA | 18 | 2.32 ± 0.33 mm | NA | NA | NA | NA | NA | Baseline: 65.08 ± 2.27 18 months: 73.92 ± 2.39 | NA | NA | NA |  |
|  |  |  |  |  |  | _ | Alloplastic | Immediate implants were placement into fresh extraction sockets combined with alloplastic material into the gap | 12 | 0 | 12 | 18 |  |  | NA | 18 | 2.49 ± 0.27 mm | NA | NA | NA | NA | NA | Baseline: 66.33 ± 2.57 18 months: 74.92 ± 1.38 | NA | NA | NA |  |
|  | **Jung et al. (2009)** | University; Single center | Switzerland | Implant placement in healed sites | RCT | rhBMP-2 | DBBM particles | At implant placement, bone defects were treated with DBBM with rhBMP-2 and a collagen membrane | 11 | 60 | 10 | 60 | •RX •Questionnaire | MBL | •Mesial: 1.33 ± 0.39 mm •Distal: 1.28 ± 0.24 mm | 60 | •Mesial: 1.36 ± 0.43 mm •Distal: 1.39 ± 0.43 mm. | •Mesial: -0.07 ± 0.31 mm •Distal: -0.11 ± 0.31 mm | NA | NA | NA | NA | NA | NA | •VAS: 57.5 ± 17.8% •Perform adequate oral hygiene: 54.5 ± 21.7% | •One patient complained about pain, swelling and inflammation | No conflict of interest reported. The study was funded by the Clinic for Fixed and Removable Prosthodontics and Dental Material Science, University of Zurich. |
|  |  |  |  |  |  | _ | DBBM particles | At implant placement, bone defects treated with DBBM and a collagen membrane | 11 | 60 | 10 | 60 |  |  | •Mesial: 1.20 ± 0.40 mm •Distal: 1.32 ± 0.47 mm | 61 | •Mesial: 1.25 ± 0.57 mm •Distal: 1.21 ± 0.46 mm. | •Mesial: -0.03 ± 0.31 mm •Distal: +0.13 ± 0.56 mm. | NA | NA | NA | NA | NA | NA | •VAS: 54 ± 22.3% •Perform adequate oral hygiene:60.5 ± 26.6% | NA |  |

Footnotes

| β-TCP/HA | β-tricalcium phosphate/hydroxyapatite |
| --- | --- |
| DBBM | deproteinated bovine bone matrix |
| HA | hydroxyapatite |
| PRGFs | Plasma Rich in Growth Factors |
| PRP | Platelet-rich plasma |
| A-PRF | Advanced platelet-rich fibrin |
| FDBA | freeze-dried bone allograft |
| ErhBMP-2 | Escherichia coliderived rhBMP-2 |
| β-TCP | β-Tricalcium phosphate |
| PI | Plaque index |
| GI | Gingival index |
| BI | Bleeding index |
| PD | Probing depth |
| MBL | Marginal bone level ( + below the implant shoulder / - above the implant shoulder) |
| ACS | Absorbable collagen sponge |
| MSCs | mesenchymal stem cells |
| FDBA | Freeze-dried bone allograft |
| BDGF | Autologous blood-derived products |
| EMD | Enamel matrix derivative |
| DBBMC | Deproteinized bovine bone mineral with 10% collage |
| FHA | fluorohydroxyapatite |
| BFPSC | Buccal fat pad-derived stem cells |
| BMAC | Bone marrow aspirate concentrate |
| Type 1 tissue | mature bone comprising woven bone and osteocytes |
| Type 2 tissue | osteoid bone’ comprising a large number of osteoblasts and less organized fibers. |
